# Green-Synthesized *Rheum cordatum* Root-Based Carbon Nanosphere-Based ZIF-8 Nanocomposite Against MRSA: In Vitro Antioxidant, Antibacterial Activity and Gene Expression Profiling

**DOI:** 10.3390/pharmaceutics18101242

**Published:** 2026-09-30

**Authors:** Başak Bedir, Mehmet Ersatir, Mehmet Çimentepe, Özge Öztürk Çimentepe, Akın Yiğin, Metin Yildirim

**Affiliations:** 1 Department of Nutrition and Dietetics, Faculty of Health Sciences, Hakkari University, Hakkâri 30000, Türkiye; 2 Department of Chemistry, Faculty of Art and Science, Cukurova University, Adana 01330, Türkiye; 3 Department of Pharmaceutical Microbiology, Faculty of Pharmacy, Harran University, Sanliurfa 63300, Türkiye; 4 Department of Pharmacology, Faculty of Pharmacy, Harran University, Sanliurfa 63300, Türkiye; 5 Department of Genetics, Faculty of Veterinary Medicine, Harran University, Sanliurfa 63200, Türkiye; 6 Department of Biochemistry, Faculty of Pharmacy, Cukurova University, Adana 01330, Türkiye

**Keywords:** ZIF-8, MRSA, carbon nanosphere, *Rheum cordatum* L., nanocomposite

## Abstract

**Background & Aim**: The emergence of multidrug-resistant pathogens, particularly methicillin-resistant *Staphylococcus aureus* (MRSA), necessitates the development of novel antimicrobial strategies. Green synthesis approaches and composite nanomaterials have attracted considerable attention as promising alternatives to conventional antimicrobial agents. Therefore, this study aimed to synthesize a carbon nanosphere/zeolitic imidazolate framework-8 (CNS@ZIF-8) nanocomposite using *Rheum cordatum* Losinsk. root-derived carbon nanospheres and to evaluate its antioxidant, antibacterial, antibiofilm, and anti-virulence properties against MRSA and methicillin-sensitive *S. aureus* (MSSA). **Methods**: Carbon nanospheres (CNS) were green-synthesized from the roots of *Rheum cordatum* Losinsk. and subsequently integrated with ZIF-8 to fabricate the CNS@ZIF-8 nanocomposite. The synthesized materials were characterized using UV–Vis spectroscopy, Fourier-transform infrared spectroscopy (FTIR), scanning electron microscopy (SEM), energy-dispersive X-ray spectroscopy (EDX), and X-ray diffraction (XRD). Antioxidant activity was assessed using DPPH and ABTS radical scavenging assays. Antibacterial activity was evaluated by determining the minimum inhibitory concentration (MIC) and minimum bactericidal concentration (MBC). Antibiofilm activity, SEM-based bacterial morphology analysis, and quantitative real-time PCR of virulence- and resistance-associated genes (*icaA*, *dltA*, *dltB*, *mepA*, and *norA*) were also performed. **Results**: Characterization analyses confirmed the successful synthesis of the CNS@ZIF-8 nanocomposite. CNS@ZIF-8 exhibited antibacterial activity against both MRSA and MSSA, with MIC and MBC values of 128 and 256 µg/mL, respectively. SEM analysis revealed severe membrane disruption and morphological damage in treated bacterial cells. Gene expression analysis demonstrated significant downregulation of biofilm formation-, cell wall modification-, and efflux pump-associated genes. CNS@ZIF-8 also showed enhanced antioxidant activity compared with pristine ZIF-8, with IC_50_ values of 45.1 ± 0.55 µg/mL (DPPH) and 9.1 ± 0.44 µg/mL (ABTS), whereas ZIF-8 exhibited IC_50_ values of 149.2 ± 0.32 and 22.3 ± 0.31 µg/mL, respectively. However, CNS alone displayed the strongest radical scavenging activity. The antioxidant standard butylated hydroxytoluene (BHT) exhibited IC_50_ values of 23.4 ± 0.42 µg/mL (DPPH) and 26.3 ± 0.63 µg/mL (ABTS). **Conclusion**: The CNS@ZIF-8 nanocomposite demonstrated promising antioxidant, antibacterial, antibiofilm, and anti-virulence activities against *S. aureus*, particularly MRSA. These findings suggest that CNS@ZIF-8 represents a multifunctional nanomaterial with potential for the development of alternative therapeutic strategies against multidrug-resistant bacterial infections.

## 1. Introduction

The global escalation of antimicrobial resistance (AMR) represents one of the most urgent threats to modern healthcare systems since it severely limits the efficacy of conventional antibiotic therapies [1]. Among multidrug-resistant pathogens, MRSA is particularly concerning due to its high virulence, adaptability, and capacity for persistent biofilm-associated infections [2]. At the molecular level, MRSA pathogenicity and resistance are associated with multiple mechanisms involving β-lactam resistance, biofilm formation, quorum sensing regulation, cell envelope modification, and multidrug efflux systems [3]. MRSA is a significant clinical challenge in both hospital and community settings due to biofilm formation and the expression of biofilm-associated genes (*icaA*), cell envelope modification-associated genes of the *dlt* operon, and efflux pump-related genes (*norA* and *mepA*). These factors all play a role in enhancing the bacterial survival, making it more tolerant to antibiotics, improving its ability to stick to surfaces, and contributing to the development of chronic infections [4,5].

Nanotechnology-based antimicrobial platforms have emerged as promising alternatives to conventional antibiotic therapies due to the increasing therapeutic limitations associated with multidrug-resistant pathogens such as MRSA [6]. A wide range of nanomaterials, including metal nanoparticles, carbon-based nanostructures, and metal–organic frameworks (MOFs), exhibit antibacterial effects through mechanisms such as reactive oxygen species (ROS) generation, membrane disruption, enzyme inhibition, and metal ion release. ROS-mediated oxidative stress induces lipid peroxidation, DNA fragmentation, and protein denaturation, ultimately leading to bacterial cell death [7,8]. Nevertheless, despite their potent antimicrobial activity, many nanomaterials still face important limitations related to cytotoxicity, environmental persistence, and limited biological selectivity [6].

Zeolitic imidazolate framework-8 (ZIF-8) is an intriguing material made up of Zn^2+^ ions linked with 2-methylimidazole ligands. It has caught the attention of researchers in the field of metal–organic frameworks (MOFs) due to its impressive structural stability, high porosity, and unique ability to degrade in response to pH changes [9].

Researchers are increasingly using carbon-based nanomaterials as strengthening components in hybrid nanoplatforms to improve the physicochemical stability and antibacterial efficacy of MOF-based systems. This strategy seeks to improve both functional synergy and structural integrity. Carbon-based nanomaterials have attracted considerable attention due to their tunable physicochemical properties, high surface area, and excellent biocompatibility [10,11]. CNS constitute a distinct class of zero-dimensional carbon architectures with enhanced structural stability compared to carbon quantum dots (CQDs) [12,13]. Their comparatively larger size may provide improved mechanical stability and facilitate integration with MOF structures [14,15].

CNS-based hybrid systems have been reported to significantly enhance antibacterial efficiency through synergistic interactions with metal ions and MOF frameworks. In these systems, CNS improve electronic conductivity and structural stability, while ZIF-8 ensures sustained Zn^2+^ release, resulting in enhanced antibacterial efficiency and physicochemical stability [16]. Despite these promising improvements in performance, the design and synthesis of such hybrid nanostructures increasingly require consideration of sustainability and environmental impact to ensure their safe and scalable development.

*Rheum cordatum* Losinsk is known to contain phenolic compounds, flavonoids, and anthraquinone derivatives, which may contribute to the reduction and stabilization processes involved in carbonization and nanoparticle formation [17]. These phytochemicals facilitate the formation of stable CNS under mild synthesis conditions through their role as natural capping and stabilizing agents in nanoparticle formation processes [18]. In this regard, *Rheum cordatum* represents a promising phytochemical resource due to its rich phenolic and anthraquinone composition, which may facilitate both carbonization and nanocomposite stabilization during green nanocomposite fabrication [19,20]. However, despite growing interest in plant-mediated nanomaterials for antimicrobial applications, the development of *Rheum cordatum*-mediated CNS@ZIF-8 hybrid nanocomposites and the investigation of their gene-level antibacterial mechanisms against MRSA remain insufficiently explored. This gap is particularly significant since it is necessary to comprehend molecular mechanisms in order to clarify the development of such nanocomposites as next-generation antimicrobial agents and to explain how they disrupt bacterial survival pathways [21].

In order to fill this gap, the current study reports the green synthesis of a ZIF-8 nanocomposite based on CNS mediated by *Rheum cordatum* root extract. A system integrating the structural advantages of CNS with the functional properties of ZIF-8 was developed to achieve enhanced antibacterial and antioxidant performance [22,23]. The nanocomposite was evaluated against MRSA for in vitro antibacterial efficacy and antioxidant activity. In addition, gene expression profiling targeting biofilm-associated (*icaA*), cell envelope modification-associated (*dltA* and *dltB*), and multidrug efflux pump-related (*mepA* and *norA*) genes was performed to characterize the transcriptional response of MRSA to CNS@ZIF-8 exposure and to explore its potential effects on resistance- and virulence-associated pathways. [21]. Taken together, this study presents a sustainable multifunctional nanoplatform that shows promise for combating multidrug-resistant bacteria and the associated infections.

## 2. Materials and Methods

### 2.1. Plant Material

The roots of *Rheum cordatum* Losinsk. used in this study were collected from the southern foothills of the Chu-Ili Mountains in the Korday District, Zhambyl Region, Kazakhstan (43°16′42.0″ N, 74°51′23.0″ E; approximately 1200 m above sea level). The plant material was taxonomically authenticated at the Institute of Botany and Phytointroduction, Ministry of Education and Science of the Republic of Kazakhstan, and a voucher specimen was deposited under herbarium accession number 0003459. The collected roots were vacuum-dried at 45–50 °C, ground into a fine powder, and stored at Cukurova University, Adana, Türkiye, until further use.

### 2.2. Synthesis of CNS, Zif-8, and Cns@zif-8 Nanocomposite

**Figure 1:**
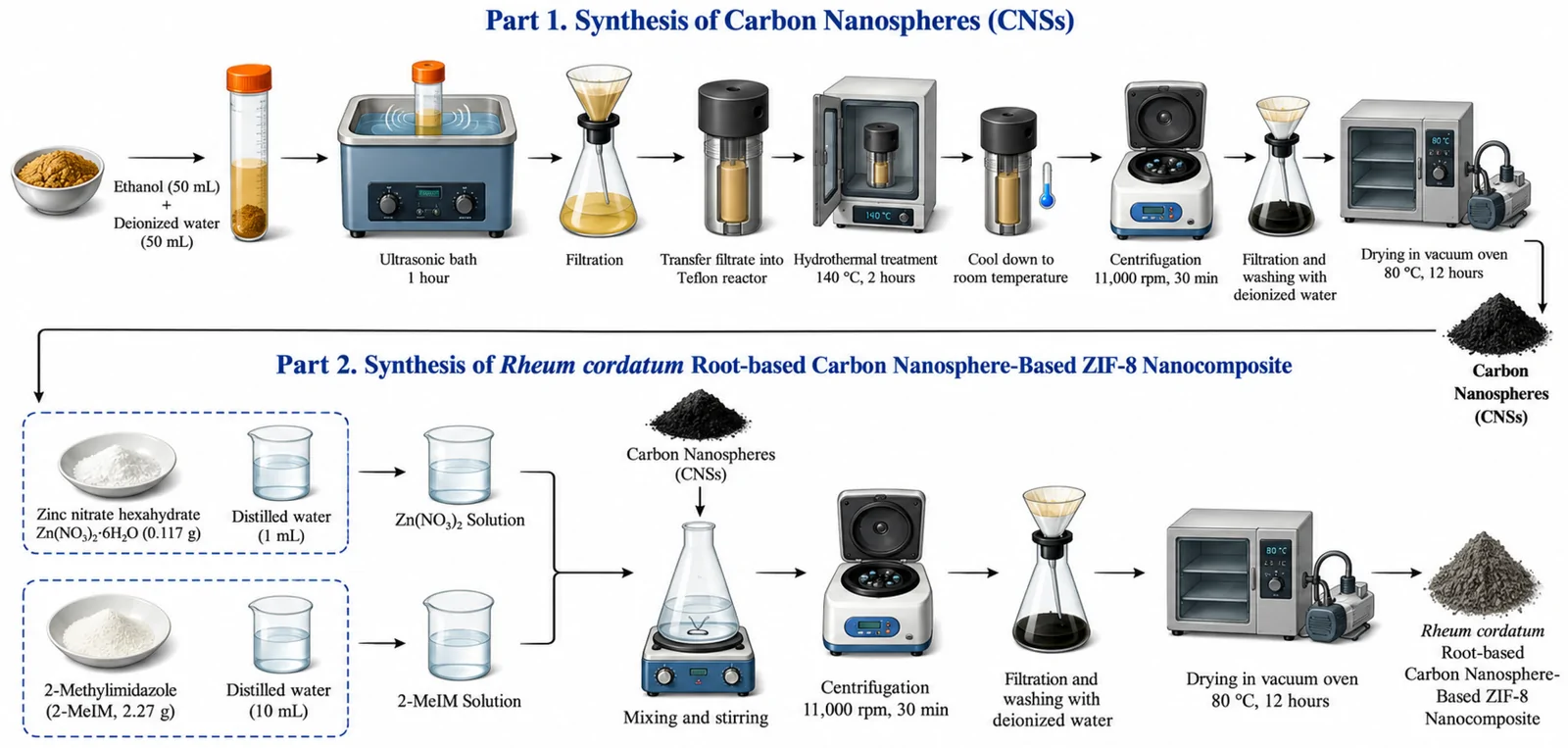
Schematic illustration of the synthesis of *Rheum cordatum* root-derived carbon nanosphere–ZIF-8 nanocomposite.

The carbon nanosphere material synthesis method was carried out according to the hydrothermal method reported in the literature [24]. Briefly, powdered *Rheum cordatum* L. roots (2.5 g) were placed into a Nalgene Teflon centrifuge tube ((Thermo Fisher Scientific, USA), and 50 mL of ethanol and 50 mL of deionized water were added. The resulting mixture was kept in an ultrasonic bath for 1 h, after which it was filtered. The filtrate was transferred into a a PTFE-lined stainless-steel container (Parr 50 ml) under autogenous pressure (Teflon reactor, Parr Instrument Company, USA) and heated at 140 °C for 2 h. At the end of the process, the mixture was cooled to room temperature, and the obtained solid–liquid mixture was centrifuged at 11,000 rpm for 30 min. The precipitated solid was filtered, washed several times with deionized water, and dried in a vacuum oven at 80 °C for 12 h (Figure 1).

### 2.3. Characterization of the Synthesized CNS, ZIF-8, and CNS@ZIF-8 Nanocomposite

#### 2.3.1. Fourier Transform Infrared Spectroscopy (FTIR)

FTIR was performed to identify the functional groups of the synthesized structures. FTIR spectra were recorded over the range of 400–4000 cm^−1^.

#### 2.3.2. X-Ray Diffraction (XRD)

The crystalline structures of the synthesized materials were characterized using an X-ray diffractometer (PANalytical EMPYREAN XRD, Malvern Panalytical, Almelo, The Netherlands). The diffraction patterns were recorded using Cu Kα radiation (Kα1 = 1.54060 Å and Kα2 = 1.54443 Å) at an operating voltage of 45 kV and a current of 40 mA. Measurements were performed in continuous scanning mode over a 2θ range of 10.00–80.00°, with a step size of 0.013° 2θ and a scan step time of 73.695 s. A fixed divergence slit of 1.0° was used, and the measurements were conducted at 25 °C. XRD peak positions were reported to two decimal places in accordance with the acquisition parameters to avoid implying unjustified analytical precision.

#### 2.3.3. Field Emission Scanning Electron Microscopy (FESEM) and Energy Dispersive X-Ray Spectroscopy (EDX)

The surface morphology of the synthesized particles was examined using Field Emission Scanning Electron Microscopy (FESEM) (FEI Quanta 650 Field Emission SEM, Thermo Fisher Scientific, Hillsboro, OR, USA). Prior to imaging, the samples were sputter-coated with a thin layer of gold to improve conductivity. The elemental composition of the synthesized particles was determined using EDX attached to the FESEM instrument. Particle sizes were quantitatively analyzed from SEM micrographs using ImageJ 1.54 g software (National Institutes of Health, USA). A total of 40 individual particles were measured from multiple independent fields of view for each material, and the results were expressed as mean ± SD, together with the corresponding size range and particle-size distribution.

#### 2.3.4. UV–Visible Spectroscopy (UV–Vis)

UV–Visible absorption spectra were recorded using a Shimadzu UV-1280 UV–Vis spectrophotometer (Shimadzu Corporation, Kyoto, Japan) over the wavelength range of 200–800 nm.

### 2.4. Microbiological Evaluations

#### 2.4.1. Agar Well Diffusion Assay

The antimicrobial activities of CNS, ZIF-8, and CNS@ZIF-8 nanoformulations were evaluated in vitro using the agar well diffusion method as previously described [25]. Bacterial strains were prepared from fresh 18–24 h cultures and suspended in sterile 0.85% NaCl solution. The turbidity of the bacterial suspensions was adjusted to the 0.5 McFarland standard, corresponding to approximately 1–2 × 10^8^ CFU/mL. The standardized inocula were uniformly spread onto the surface of Mueller–Hinton agar plates using sterile cotton swabs.

The inoculated plates were allowed to stand at room temperature for 10–15 min, after which wells with a diameter of 7 mm were aseptically punched into the agar using a sterile cork borer. Subsequently, 100 μL of each stock solution (8 mg/mL) was dispensed into the respective wells. A 30 μg cefoxitin (FOX) disk (Oxoid, UK) was used as the reference antibiotic control. The plates were then incubated at 37 °C for 18–24 h. Following incubation, the diameters of the inhibition zones were measured in millimeters and recorded as indicators of antimicrobial activity.

#### 2.4.2. Determination of Minimum Inhibitory Concentration (MIC) and Minimum Bactericidal Concentration (MBC)

The antibacterial activities of CNS, ZIF-8, and CNS@ZIF-8 nanoformulations against methicillin-susceptible *Staphylococcus aureus* (MSSA, ATCC 25923) and methicillin-resistant *Staphylococcus aureus* (MRSA, ATCC 43300) were evaluated using the resazurin microdilution assay (REMA) [26]. In this study, CNS, ZIF-8, and CNS@ZIF-8 nanoformulations were prepared at concentrations ranging from 2 to 1024 µg/mL and added to the respective wells. The bacterial inocula were adjusted to the 0.5 McFarland standard, and appropriate dilutions were performed to obtain a final bacterial density of 1 × 10^5^ CFU/mL in each well. The prepared microplates were incubated at 37 °C for 18–24 h.

Following incubation, sterile resazurin solution (0.01%, *w*/*v*) was added to each well, and the plates were further incubated. Changes in color resulting from bacterial metabolic activity were evaluated, where the development of a pink color indicated bacterial growth, while retention of the blue color indicated inhibition of bacterial proliferation. Accordingly, the lowest concentration at which the blue color was maintained was recorded as the MIC value [26,27].

For the determination of the minimum bactericidal concentration (MBC), aliquots were taken from wells showing no visible bacterial growth and spread onto Mueller–Hinton Agar plates, followed by incubation under appropriate conditions. After incubation, the lowest concentration of the tested nanoformulations that resulted in a 99.9% reduction in bacterial viability compared with the initial inoculum was recorded as the MBC value. All experiments were performed in triplicate using three independent biological replicates to ensure the reliability and reproducibility of the results.

#### 2.4.3. Time–Kill Assay

The time-dependent bactericidal activities of CNS, ZIF-8, and CNS@ZIF-8 against the MRSA strain were evaluated using the time–kill assay. Bacterial suspensions were prepared from freshly grown cultures and adjusted to the 0.5 McFarland standard. The suspensions were subsequently diluted in Mueller–Hinton Broth (MHB) to obtain a final bacterial concentration of approximately 1 × 10^6^ CFU/mL.

CNS, ZIF-8, and CNS@ZIF-8 were added to the test medium at concentrations corresponding to their previously determined 1 × MIC and 2 × MIC values, and the mixtures were incubated at 37 °C. To assess time-dependent changes in bacterial viability, aliquots were collected after 4, 8, and 24 h of incubation. The collected samples were subjected to serial ten-fold dilutions in sterile physiological saline, and appropriate dilutions were plated onto Mueller–Hinton Agar (MHA) plates. Following incubation at 37 °C for 18–24 h, the resulting colonies were counted, and bacterial viability was expressed as log_10_ CFU/mL. The bacterial counts obtained from the treated groups were compared with those of the untreated growth control. A reduction of ≥3 log_10_ CFU/mL relative to the initial inoculum or the growth control was considered indicative of bactericidal activity. All experiments were performed in triplicate with three independent biological replicates. The obtained data were analyzed by constructing time–kill curves to evaluate the kinetics of bacterial reduction over time [28].

#### 2.4.4. Synergistic Effect of Zif-8 and CNS Determined by Checkerboard Assay

The antimicrobial interaction between CNS and ZIF-8 was evaluated using the broth microdilution checkerboard method with slight modifications to a previously published procedure [29]. Serial two-fold dilutions of CNS and ZIF-8 were prepared to obtain final concentrations ranging from 2 to 1024 µg/mL. A standardized bacterial inoculum was added to each well, resulting in a final cell density of 5 × 10^5^ CFU/mL. The microplates were incubated at 37 °C for 18–24 h. After incubation, bacterial viability was assessed using the resazurin-based microtiter assay, and the MIC values for each ZIF-8–CNS combination were determined. The interaction between the two agents was evaluated by calculating the fractional inhibitory concentration index (FICI) using the following equation:

FICI = (MIC of ZIF-8 s in combination/MIC of ZIF-8 alone) + (MIC of CNS in combination/MIC of CNS alone). The interactions were classified according to the calculated FICI values as follows: synergistic (FICI ≤ 0.5), additive or indifferent (0.5 < FICI ≤ 4), and antagonistic (FICI > 4).

#### 2.4.5. Antibiofilm Activity

The inhibitory effects of CNS, ZIF-8, and CNS@ZIF-8 on MRSA biofilm formation were evaluated using the crystal violet staining method [30]. Fresh MRSA cultures were grown in Tryptic Soy Broth (TSB) and distributed into 96-well microplates to promote biofilm formation. The plates were incubated at 37 °C for 24 h to allow biofilm development. Following incubation, the culture medium was removed, and the wells were gently washed with sterile phosphate-buffered saline (PBS) to eliminate non-adherent cells.

Subsequently, CNS, ZIF-8, and CNS@ZIF-8 nanoformulations were prepared at concentrations corresponding to 2 × MIC, 1 × MIC, and ½ × MIC and added to the respective wells. The plates were then incubated for an additional 24 h at 37 °C. At the end of the incubation period, planktonic cells were removed, and the attached biofilms were fixed by adding 99% methanol to each well and allowing the plates to stand for an appropriate period. After fixation, the plates were air-dried and stained with 0.5% crystal violet solution to quantify biofilm biomass.

Excess stain was removed by washing the wells with PBS, and the plates were allowed to dry at room temperature. The crystal violet bound to the biofilm matrix was subsequently solubilized using 95% ethanol. After a 10 min incubation period, absorbance values were measured at 570 nm using a microplate reader (Multiskan SkyHigh, Thermo Fisher Scientific, Singapore). All experiments were performed in triplicate with three independent biological replicates, and biofilm inhibition percentages were calculated based on the obtained optical density values.

#### 2.4.6. Bacterial Morphology

Morphological alterations induced by CNS@ZIF-8 in MRSA cells were examined using scanning electron microscopy (SEM, ZEISS EVO 50, Jena Germany). Briefly, 1 mL of bacterial suspension (1 × 10^5^ CFU/mL) was inoculated into each well of a 12-well plate, followed by the addition of 1 mL of the test compound at a concentration corresponding to 2 × MIC. The samples were incubated at 37 °C for 24 h. Wells containing only culture medium and bacterial suspension without treatment served as the untreated control. Following incubation, the samples were fixed overnight at 4 °C using 3% (*v*/*v*) glutaraldehyde solution. The fixed specimens were then dehydrated through a graded ethanol series (50–100%). After dehydration, the samples were dried and coated with a thin layer of gold using a sputter-coating technique to ensure surface conductivity. Finally, the bacterial specimens were visualized using SEM, and the resulting morphological and structural alterations were recorded and compared with those of the untreated control group [31].

### 2.5. Gene Expression Analysis

The effects of CNS@ZIF-8 on the expression levels of the target genes *icaA*, *dltA*, *dltB*, *mepA*, and *norA* in MRSA were investigated, with *16S rRNA* serving as the reference gene (Table 1) [32,33,34,35]. For gene-expression analysis, MRSA cultures were adjusted to a bacterial suspension of 1 × 10^5^ CFU/mL and exposed to CNS@ZIF-8 at the MIC concentration (128 µg/mL) for 24 h prior to RNA extraction. The MIC concentration was selected to reduce the potential confounding effects associated with extensive bacterial killing and to allow assessment of transcriptional responses under inhibitory exposure conditions. The qRT-PCR experiment was specifically designed to characterize the transcriptional response of MRSA to the final CNS@ZIF-8 nanocomposite. Following treatment, total RNA was extracted using the High Pure RNA Isolation Kit (Roche, Mannheim, Germany) according to the manufacturer’s instructions. RNA purity and concentration were evaluated using a NanoDrop spectrophotometer (DeNovix DS-11, DeNovix Inc., Wilmigton, DE, USA). The RNA concentrations were 276, 312, and 341 ng/µL, respectively, with A260/A280 ratios ranging from 1.92 to 2.01. Prior to cDNA synthesis, RNA samples were normalized to 200 ng/µL to ensure equal RNA input across samples. Complementary DNA (cDNA) was synthesized from the isolated RNA samples using the Transcriptor First Strand cDNA Synthesis Kit (Roche, Mannheim, Germany; Cat. No. 04896866001). Reverse transcription was performed at 25 °C for 10 min, followed by 55 °C for 30 min and 85 °C for 5 min. Quantitative real-time PCR (qRT-PCR) analyses were subsequently carried out using LightCycler^®^ FastStart DNA Master SYBR Green I (Roche, Mannheim, Germany) on a Rotor-Gene Q Real-Time PCR System (Qiagen, Germany). The amplification protocol consisted of an initial denaturation step at 95 °C for 5 min, followed by 40 cycles of denaturation at 95 °C for 5 s and primer annealing/extension at the respective optimal annealing temperatures for 30 s. The standard curve analysis demonstrated good linearity (R^2^ = 0.991), with a slope of −3.218 and an amplification efficiency of approximately 105%, indicating acceptable and reliable qRT-PCR amplification performance.

Relative gene expression levels were calculated using the 2^−ΔΔCt^ method [36]. All experiments were performed in triplicate using three independent biological replicates to ensure the reliability and reproducibility of the results.

### 2.6. Antioxidant Activity

#### 2.6.1. ABTS Radical Cation Scavenging Activity

The antioxidant potentials of CNS, ZIF-8, and CNS@ZIF-8 formulations were evaluated using the ABTS^+^ (2,2′-azino-bis(3-ethylbenzothiazoline-6-sulfonic acid)) radical cation scavenging assay. The experimental procedure was performed with minor modifications based on the method previously reported by Amangeldinova et al. [19].

Briefly, different concentrations of CNS, ZIF-8, and CNS@ZIF-8 nanoformulations were prepared. For the assay, 0.5 mL of each sample solution was mixed with 2.5 mL of ABTS working solution and vortexed to ensure complete homogenization. The reaction mixtures were incubated for 30 min at room temperature under dark conditions. Following incubation, absorbance values were measured at 734 nm using a UV–Vis spectrophotometer (UV-1800, Shimadzu Corporation, Kyoto, Japan). Butylated hydroxytoluene (BHT) was used as the reference antioxidant, and standard solutions were prepared at concentrations ranging from 5 to 80 µg/mL. ABTS radical cation scavenging activity was calculated as percentage inhibition based on the changes in absorbance values. In addition, IC_50_ values (µg/mL), defined as the concentration required to scavenge 50% of ABTS radicals, were determined and used for comparative evaluation of antioxidant activity.

#### 2.6.2. DPPH Free Radical Scavenging Activity

The free radical scavenging capacities of CNS, ZIF-8, and CNS@ZIF-8 formulations were determined using the 2,2-diphenyl-1-picrylhydrazyl (DPPH) assay. The experimental procedure was carried out with minor modifications according to the protocol described by Amangeldinova et al. [19].

Briefly, 0.5 mL of freshly prepared 0.1 mM DPPH solution was added to 1.5 mL of sample solutions prepared at various concentrations. The resulting mixtures were incubated for 30 min at room temperature in the dark. After incubation, the absorbance values of the reaction mixtures were measured spectrophotometrically at 517 nm. BHT was employed as the reference antioxidant, and standard solutions were prepared within the concentration range of 5–80 µg/mL. DPPH radical scavenging activity was calculated as percentage inhibition using the absorbance values of the sample and control groups. All experiments were performed in triplicate with three independent biological replicates, and IC_50_ values were calculated from the obtained data. Lower IC_50_ values were interpreted as indicative of higher antioxidant capacity.

### 2.7. Biocompatibility Test

The cytocompatibility of CNS@ZIF-8 formulations was determined by means of an MTT-based cell viability assay performed on NIH/3T3 mouse fibroblasts (ATCC CRL-1628). Briefly, NIH/3T3 cells were distributed into 96-well culture plates at a density of 2 × 10^4^ cells per well and maintained under standard culture conditions until adequate attachment was achieved. Subsequently, CNS@ZIF-8 formulations were introduced into the wells at concentrations ranging from 8 to 128 μg/mL, while cells receiving no treatment were used as the control group. Following a 24 h exposure period at 37 °C in a humidified atmosphere containing 5% CO_2_, cell viability was quantified using the MTT assay. For this purpose, MTT reagent was added to each well at a final concentration of 0.5 mg/mL, followed by a further 3 h incubation. The metabolically generated formazan products were then solubilized by adding 100 μL of DMSO. Absorbance values were subsequently measured at 570 nm with a microplate reader. Each experimental condition was analyzed using three independent replicates.

## 3. Result and Discussion

### 3.1. Characterization of the Synthesized CNS, ZIF-8, and CNS@ZIF-8 Nanocomposite

#### 3.1.1. UV–Vis Absorption Analysis

**Figure 2:**
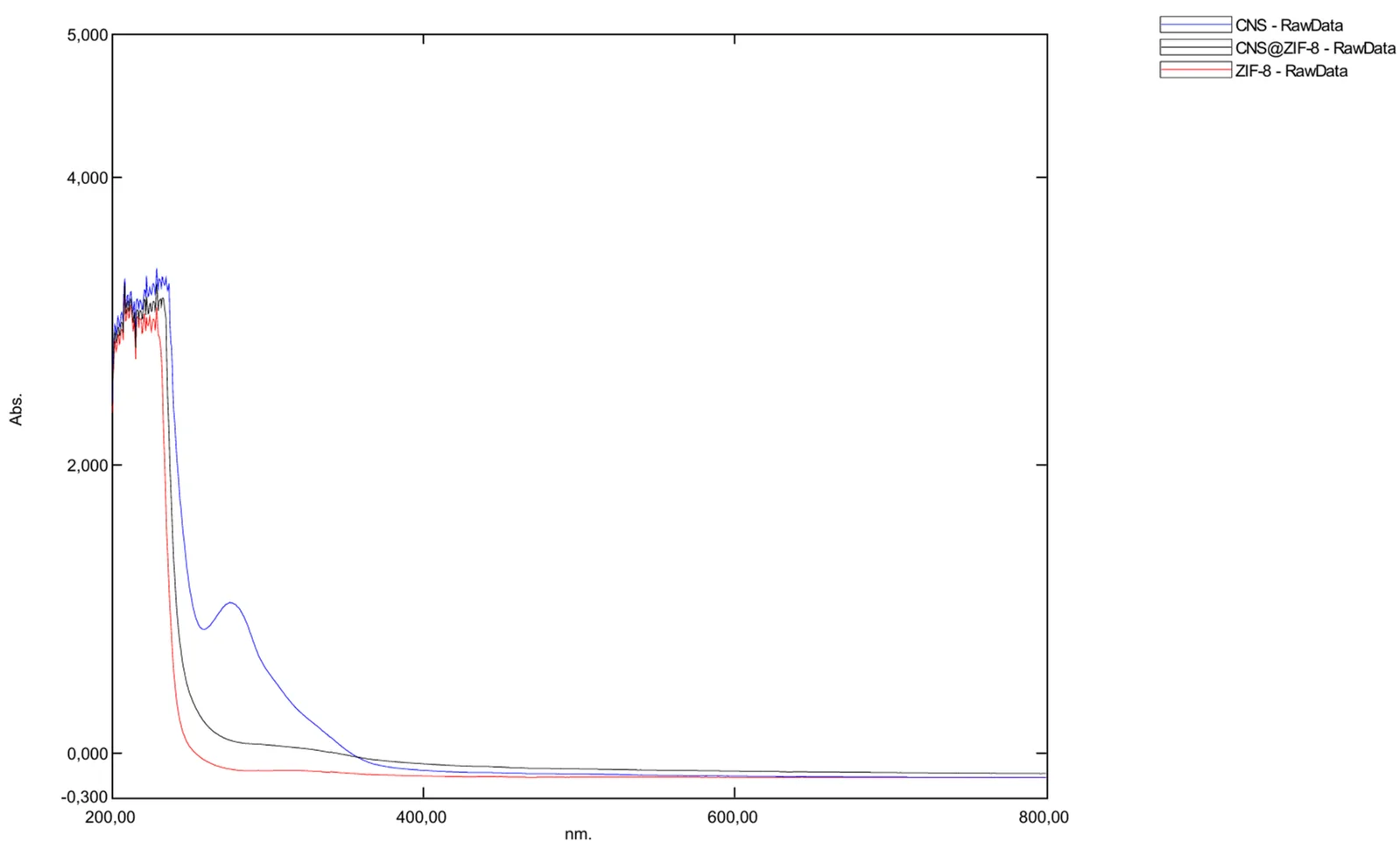
Physicochemical characterization of CNS, ZIF-8, and the CNS@ZIF-8 nanocomposite. UV–Vis absorption spectra of CNS, ZIF-8, and CNS@ZIF-8, recorded in the wavelength range of 200–800 nm to evaluate the optical properties of the synthesized materials.

The UV–Vis absorption spectra of the synthesized particles were investigated to evaluate their optical properties. The UV–Vis spectrum of CNS exhibited a characteristic absorption peak at approximately 285 nm, which can be attributed to the π→π* electronic transitions of aromatic C=C bonds present in the carbonaceous structure. In contrast, pure ZIF-8 displayed a characteristic absorption peak around 220 nm, corresponding to ligand-centered electronic transitions within the imidazolate framework (Figure 2). Furthermore, upon the formation of the CNS@ZIF-8 nanocomposite, noticeable changes in the absorption profile, including variations in peak intensity and position, were observed compared to the individual components. These spectral modifications suggest strong interactions between CNS and the ZIF-8 framework. The observed shifts and broadening of the absorption bands provide clear evidence for the successful incorporation of CNS into the ZIF-8 structure and confirm the successful synthesis of the CNS@ZIF-8 nanocomposite. Moreover, the enhanced absorption behavior of the nanocomposite compared to pristine ZIF-8 indicates the contribution of the carbonaceous component to the optical properties of the hybrid material.

#### 3.1.2. FTIR Analysis

**Figure 3:**
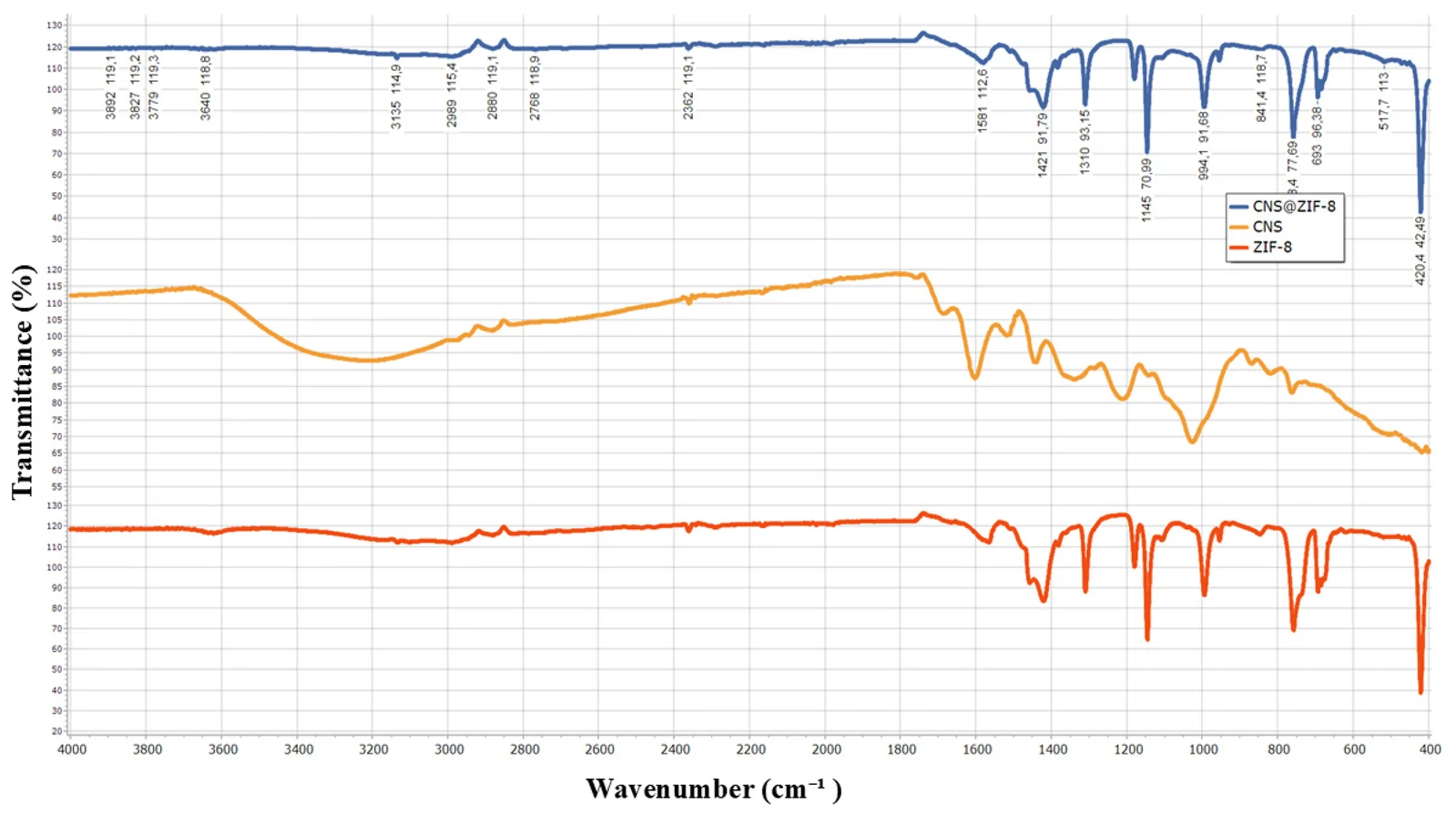
FTIR spectra of CNS, ZIF-8, and CNS@ZIF-8 recorded in the spectral range of 4000–400 cm^−1^ to identify the characteristic functional groups and to confirm the successful formation of the nanocomposite.

FTIR analysis was conducted to investigate the structural characteristics of CNS, ZIF-8, and the CNS@ZIF-8 composite. The FTIR spectrum of CNS exhibited a broad absorption band in the range of 3200–3400 cm^−1^, corresponding to the stretching vibrations of hydroxyl (–OH) groups present on the carbon surface. Characteristic absorption bands of ZIF-8 were observed at 1581, 1422, 1310, 1145, 994, 759, and 693 cm^−1^, which are attributed to the vibrational modes of the 2-methylimidazole linker. In addition, the absorption band at approximately 420 cm^−1^ was assigned to the Zn–N stretching vibration, confirming the formation of the ZIF-8 framework (Figure 3). The FTIR spectrum of the CNS@ZIF-8 composite retained the characteristic bands of both CNS and ZIF-8, indicating the successful formation of the composite structure. Furthermore, the preservation of the Zn–N vibrational band and imidazole-related peaks suggests that the crystalline framework of ZIF-8 remained intact after composite preparation. No significant new absorption bands were observed, implying that the composite was formed without substantial alteration of the chemical structures of the individual components.

#### 3.1.3. FESEM Analysis

**Figure 4:**
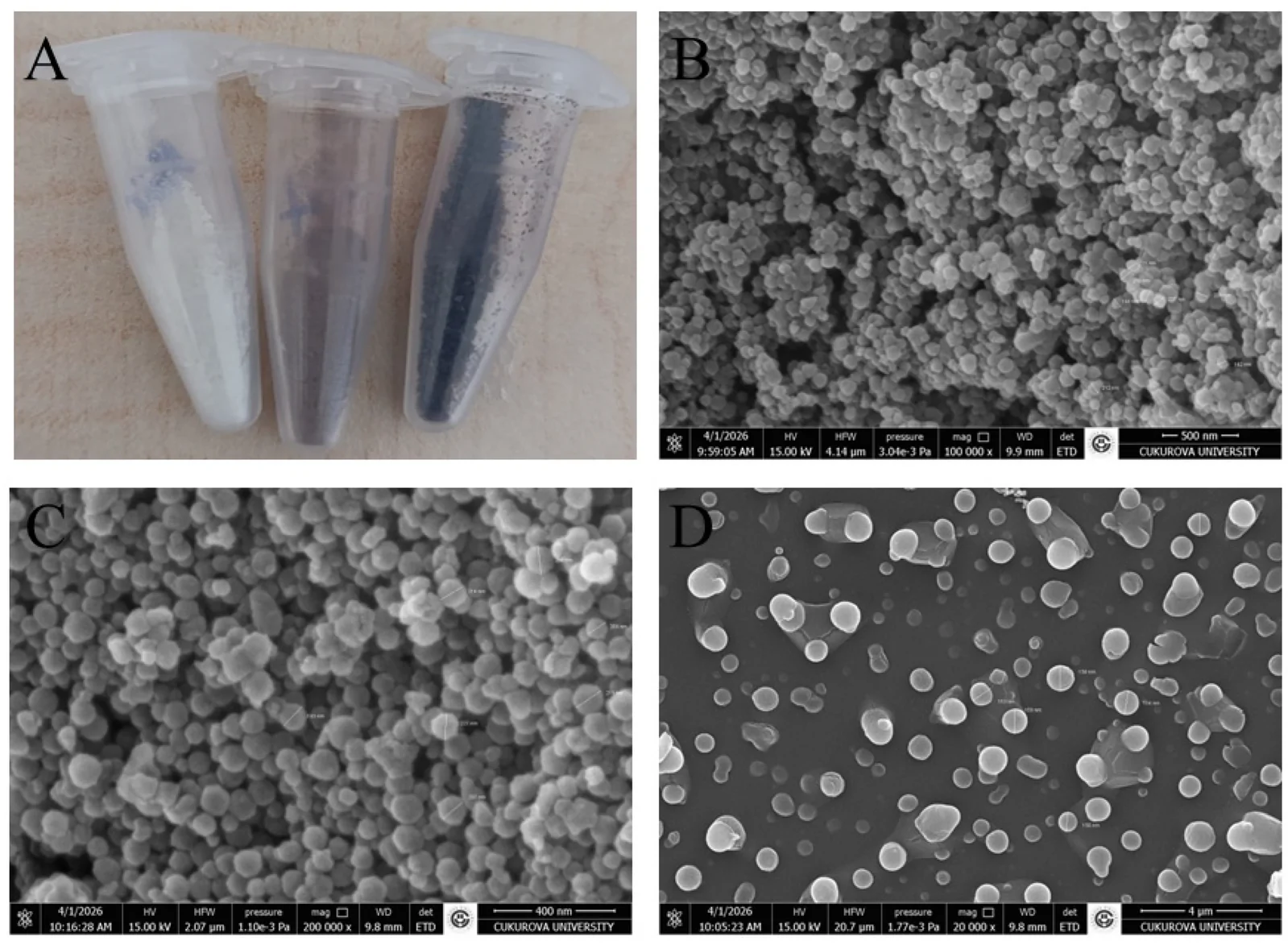
Macroscopic appearance and morphological characterization of the synthesized materials. (**A**) Digital photograph of the synthesized CNS, ZIF-8, and CNS@ZIF-8 powders. (**B**) SEM micrograph of pristine ZIF-8 showing its characteristic rhombic dodecahedral crystal morphology. (**C**) SEM micrograph of the CNS@ZIF-8 nanocomposite showing the incorporation of carbon nanospheres within the ZIF-8 framework and the resulting surface morphology. (**D**) SEM micrograph of CNS showing the morphology of the green-synthesized carbon nanospheres.

SEM analysis revealed that pristine CNS exhibited a spherical morphology with an average particle size of approximately 154.8 ± 3.1 nm. Although the majority of CNS particles were within this size range, particles with different dimensions were also observed, indicating a relatively broad size distribution. The average particle sizes of ZIF-8 and CNS@ZIF-8 were determined as 213.7 ± 5.1 nm and 211.7 ± 4.8 nm, respectively. Following the incorporation of ZIF-8, the CNS surface became densely covered with quasi-spherical nanoparticles, resulting in a rougher and more compact morphology. The comparable particle sizes observed for ZIF-8 and CNS@ZIF-8 suggest the successful growth of ZIF-8 crystals on the CNS surface without significant alteration of the crystal dimensions, confirming the formation of the CNS@ZIF-8 nanocomposite (Figure 4).

#### 3.1.4. EDX Analysis

**Figure 5:**
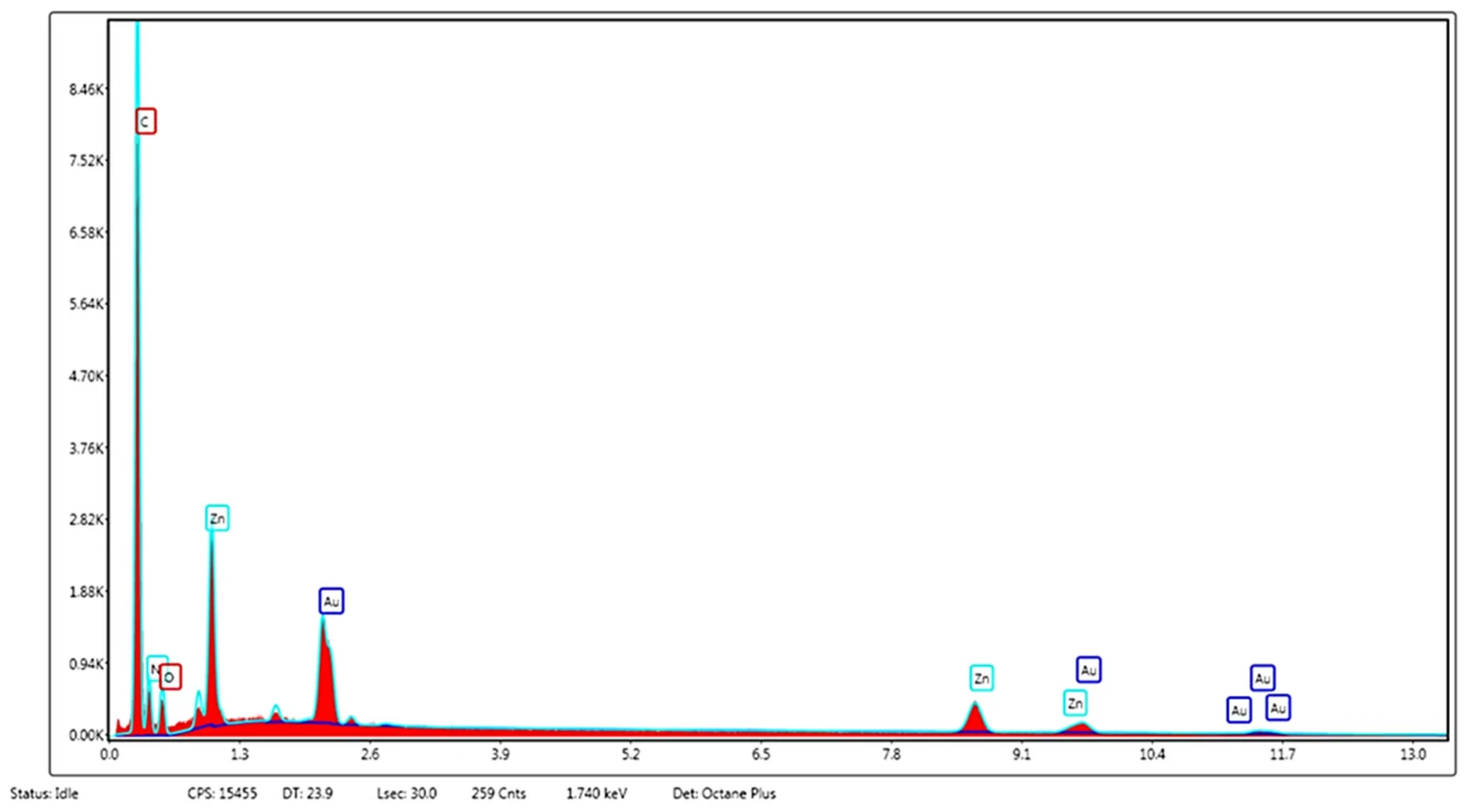
EDX analysis of CNS@ZIF-8. The *X*-axis represents energy levels in keV, while the *Y*-axis shows the intensity in counts.

**Figure 6:**
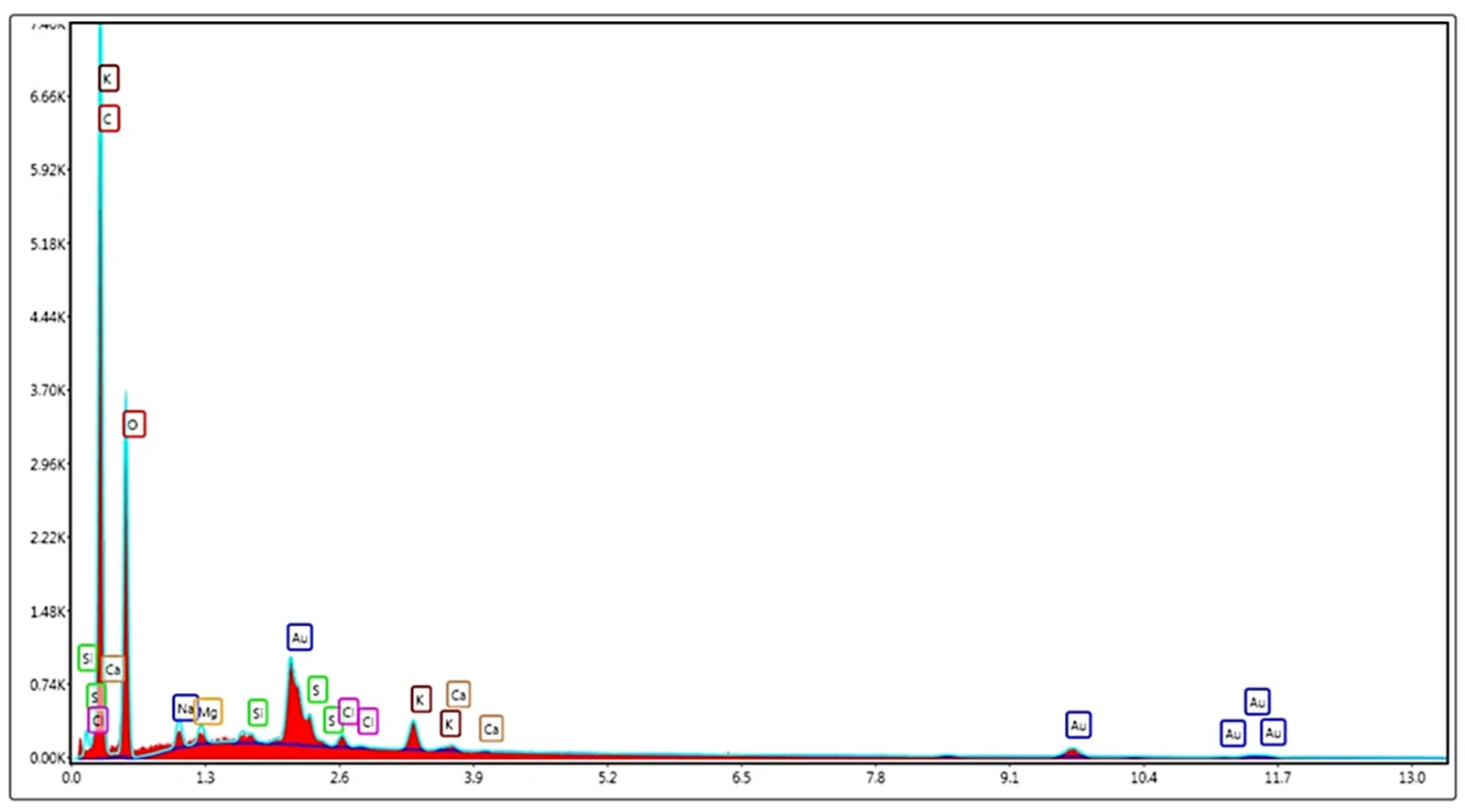
EDX analysis of CNS. The *X*-axis represents energy levels in keV, while the *Y*-axis shows the intensity in counts.

EDX analysis was performed to evaluate the elemental composition of CNS and CNS@ZIF-8. As shown in Figure 5, the CNS@ZIF-8 composite was predominantly composed of C, N, O, and Zn, with weight percentages of 52.3%, 21.4%, 8.1%, and 7.2%, respectively. The presence of Zn and the substantial N content are consistent with the incorporation of the ZIF-8 phase into the CNS-based composite. In contrast, CNS was predominantly composed of carbon (50.5 wt%) and oxygen (35.7 wt%), consistent with the carbonaceous and oxygen-containing surface functionalities of the hydrothermally derived material (Figure 6). In addition to C and O, weak signals corresponding to S, Ca, Na, Mg, Si, Cl, and K were detected in the CNS spectrum. These minor signals are attributed to residual inorganic/mineral constituents originating from the plant-derived precursor and/or trace residues remaining after the synthesis process. Because these elements were present only at trace levels, they were not included in the quantitative EDX table generated for the major elemental constituents. The Au peaks observed in the spectra originate from the gold coating applied during sample preparation for electron microscopy and therefore do not represent the intrinsic elemental composition of the samples. Following ZIF-8 incorporation, the appearance of characteristic Zn and N signals together with the marked change in the elemental profile provides additional evidence for the formation of CNS@ZIF-8. These observations are consistent with the FTIR and morphological characterization results.

#### 3.1.5. XRD Analysis

**Figure 7:**
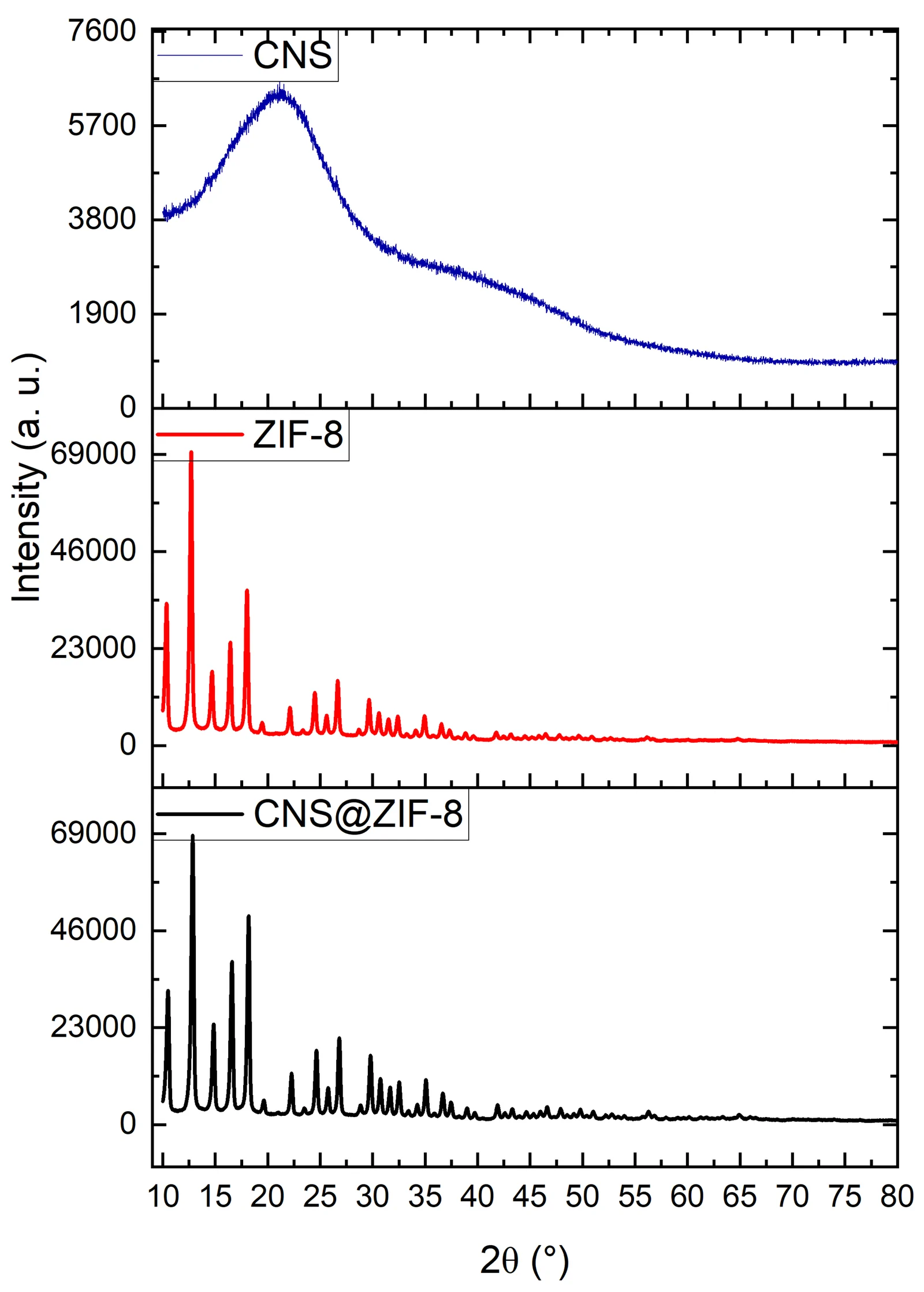
XRD patterns of CNS, pristine ZIF-8, and CNS@ZIF-8 nanocomposite. CNS exhibits a broad diffraction profile characteristic of the carbonaceous phase, whereas pristine ZIF-8 displays well-defined crystalline reflections. The CNS@ZIF-8 nanocomposite retains the principal diffraction features of ZIF-8, with slight shifts in peak positions and changes in relative peak intensities, indicating that the crystalline characteristics of ZIF-8 are largely preserved following incorporation of CNS. XRD patterns are presented over a consistent 2θ range to facilitate direct structural comparison among the materials.

The XRD patterns of CNS, ZIF-8, and CNS@ZIF-8 were analyzed to evaluate the structural characteristics of the synthesized materials (Figure 7). Pristine ZIF-8 exhibited prominent diffraction peaks at 2θ = 10.46°, 12.81°, 14.80°, 16.55°, and 18.13°, confirming its crystalline structure. In the CNS@ZIF-8 nanocomposite, the corresponding reflections were observed at 10.32°, 12.67°, 14.66°, 16.40°, and 17.98°, respectively. The preservation of these principal diffraction features indicates that the crystalline characteristics of the ZIF-8 phase were largely retained following incorporation of CNS.

Comparative analysis revealed small but systematic shifts of the principal ZIF-8-related reflections toward lower 2θ values in CNS@ZIF-8. For example, the reflections at 10.46°, 12.81°, 14.80°, 16.55°, and 18.13° shifted to 10.32°, 12.67°, 14.66°, 16.40°, and 17.98°, respectively, corresponding to Δ2θ values of approximately −0.13° to −0.15°. These slight shifts may be associated with interactions between CNS and the ZIF-8 phase and/or minor changes in the local structural environment during composite formation. Nevertheless, because the principal ZIF-8-related reflections remained clearly detectable, CNS incorporation did not result in substantial disruption of the crystalline characteristics of the ZIF-8 phase.

In contrast, CNS exhibited a simpler diffraction profile, with reflections at 2θ = 12.85°, 21.14°, and 30.34°. Diffraction features in similar regions were observed in CNS@ZIF-8 at approximately 12.67°, 20.85°, and 30.57°, respectively. The coexistence of CNS-associated diffraction features and the characteristic reflections of ZIF-8 in the composite pattern supports the formation of the CNS@ZIF-8 composite. Overall, the XRD results indicate that composite formation produced minor changes in the local structural environment while largely preserving the crystalline characteristics of the ZIF-8 phase.

### 3.2. Microbiological Assay

#### 3.2.1. Evaluation of Agar Well Diffusion Method

**Figure 8:**
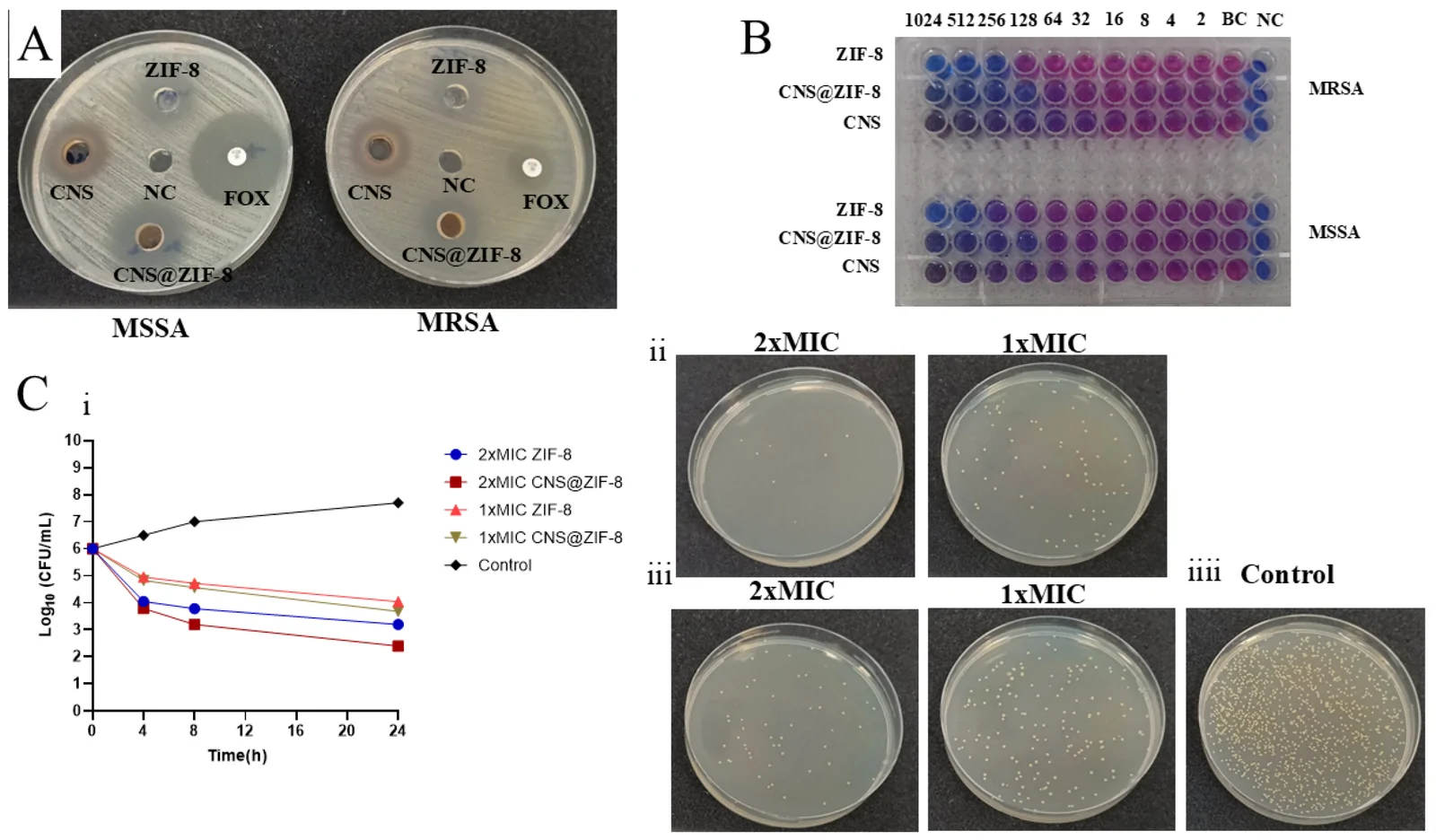
Antibacterial activity of CNS, ZIF-8, and the CNS@ZIF-8 nanocomposite against MSSA and MRSA. (**A**) Agar well diffusion assay showing the antibacterial activity of CNS, ZIF-8, and CNS@ZIF-8 against MSSA and MRSA. FOX (cefoxitin) was used as the positive control, while NC represents the negative control. (**B**) Representative resazurin-based broth microdilution assay used to determine the minimum inhibitory concentration (MIC) of CNS, ZIF-8, and CNS@ZIF-8 against MRSA and MSSA. Wells are arranged according to two-fold serial dilutions ranging from 1024 to 2 µg/mL. Blue wells indicate inhibition of bacterial growth, whereas pink wells indicate metabolically active bacterial cells. BC and NC represent the bacterial growth control and negative (sterility) control, respectively. (**C**) Time–kill kinetics of CNS@ZIF-8 against MRSA. (**i**) Time–kill curve showing the bactericidal activity of CNS@ZIF-8 and pristine ZIF-8 at 1 × MIC and 2 × MIC over a 24 h incubation period. (**ii**) Representative agar plates corresponding to bacterial cultures treated with CNS@ZIF-8 at 2 × MIC and 1 × MIC after 24 h incubation. (**iii**) Representative agar plates corresponding to bacterial cultures treated with pristine ZIF-8 at 2 × MIC and 1 × MIC, together with the untreated control group, demonstrating the concentration-dependent antibacterial activity. (**iiii**) Representative agar plates corresponding to the bacterial culture control.

The agar well diffusion assay results demonstrated that the tested CNS, ZIF-8, and CNS@ZIF-8 nanoformulations exhibited varying degrees of antibacterial activity against both MRSA and MSSA strains (Figure 8A). Against the MRSA strain, the inhibition zone diameters produced by CNS, ZIF-8, and CNS@ZIF-8 were determined as 17 ± 0.47 mm, 13 ± 0.60 mm, and 16 ± 0.45 mm, respectively. The cefoxitin (FOX) disk used as the positive control generated an inhibition zone of 14 ± 0.25 mm.

For the MSSA strain, the inhibition zone diameters measured for CNS, ZIF-8, and CNS@ZIF-8 were 19 ± 0.85 mm, 15 ± 0.54 mm, and 21 ± 0.57 mm, respectively. The positive control, FOX, exhibited the strongest antibacterial activity against MSSA, producing an inhibition zone of 31 ± 0.30 mm.

In a previous study, ZIF-8 exhibited an inhibition zone diameter of 10.5 mm against *S. aureus*. Upon incorporation of 10% and 20% *Pterocarya fraxinifolia* leaf extract into the ZIF-8 structure, the inhibition zone diameters increased to 15 mm and 22 mm, respectively. These findings indicated that PF@ZIF-8 not only enhanced antibacterial activity but also exerted a synergistic effect [37]. Similarly, in the present study, the CNS@ZIF-8 composite demonstrated superior antibacterial activity against both bacterial strains compared with pristine ZIF-8. The enhanced antibacterial performance of the composite may be attributed to the synergistic interaction between the CNS matrix and the ZIF-8 framework, which potentially improved bacterial cell membrane disruption and facilitated more effective antimicrobial action.

#### 3.2.2. Determination of MIC and MBC

The antibacterial activities of CNS, ZIF-8, and CNS@ZIF-8 formulations against MRSA and MSSA were evaluated by determining their MIC and MBC values (Table 2). The obtained results demonstrated notable differences in antibacterial efficacy among the tested formulations (Figure 8B). Against the MRSA strain, CNS exhibited an MIC value of 512 µg/mL, whereas ZIF-8 showed improved antibacterial activity with an MIC value of 256 µg/mL. The CNS@ZIF-8 nanocomposite demonstrated the highest activity among the tested materials, with an MIC value of 128 µg/mL. A similar trend was observed against MSSA, with MIC values of 512, 256, and 128 µg/mL for CNS, ZIF-8, and CNS@ZIF-8, respectively. However, compared with vancomycin, which exhibited MIC values of 2 µg/mL against MRSA and 1 µg/mL against MSSA, the tested materials showed substantially lower antibacterial potency.

ZIF-8 has demonstrated antimicrobial activity against diverse bacterial and fungal pathogens, including resistant strains [38]. Its combination with conventional antibiotics may further improve antimicrobial efficacy and help reduce antibiotic exposure [39]. Green synthesis strategies using safer solvents have emerged as sustainable alternatives to conventional ZIF-8 preparation with toxic solvents such as DMF [40]. In this study, CNS@ZIF-8 was successfully synthesized using *Rheum cordatum*-derived carbon nanospheres.

Several studies have demonstrated that the incorporation of bioactive plant-derived compounds into ZIF-8 structures substantially enhances their antimicrobial efficacy. Saif et al. reported that the MIC value of *Cordia myxa* extract against *S. aureus* was 125 µg/mL, whereas the MIC value decreased to 31.25 µg/mL following incorporation into a CME@ZIF-8 nanocomposite. In the same study, ZIF-8 alone exhibited an MIC value of 62.5 µg/mL against *S. aureus* [41]. Similarly, the antibacterial activity of a PF@ZIF-8 nanocomposite synthesized using *Pterocarya fraxinifolia* leaf extract was evaluated against *S. aureus*. The MIC values of the plant extract and PF@ZIF-8 were reported as 70 and 50 µg/mL, respectively, while ZIF-8 alone exhibited an MIC value greater than 100 µg/mL [37]. Likewise, an APE@ZIF-8 nanocomposite synthesized using *Ajuga parviflora* extract demonstrated potent antibacterial activity against MRSA, with an MIC value of 61.25 µg/mL. In contrast, both the plant extract and ZIF-8 alone exhibited MIC values of 250 µg/mL [42]. Collectively, these findings indicate that loading biologically active compounds into the ZIF-8 framework significantly enhances antibacterial performance.

In our study, the incorporation of CNS into the ZIF-8 nanocarrier system significantly enhanced its antibacterial activity against both MRSA and MSSA. Notably, the CNS@ZIF-8 formulation exhibited a four-fold lower MIC value than free CNS, suggesting a substantial improvement in antibacterial potency. The enhanced activity of CNS@ZIF-8 may be attributed to the high surface area and porous structure of ZIF-8, which can facilitate more efficient interaction between the active components and bacterial cells, thereby increasing antimicrobial effectiveness. Furthermore, the comparable susceptibility profiles observed for both MRSA and MSSA suggest that the antibacterial mechanism of CNS@ZIF-8 is not markedly affected by methicillin resistance, highlighting its potential as a promising nanocomposite for the treatment of infections caused by both susceptible and resistant *S. aureus* strains.

The antibacterial activity of ZIF-8 has been attributed to several complementary mechanisms. One proposed mechanism involves the induction of reactive oxygen species (ROS), leading to oxidative damage of bacterial proteins, nucleic acids, and membrane lipids. A second mechanism is associated with the release of Zn^2+^ ions from the ZIF-8 framework, which can interfere with intracellular proteins and enzymes, disrupt metabolic pathways, and impair essential cellular functions. Moreover, Zn^2+^ ions have been reported to disturb intracellular ion homeostasis, thereby restricting bacterial proliferation. A third mechanism involves the direct interaction of ZIF-8 nanoparticles with the bacterial cell wall and membrane, resulting in membrane disruption, increased permeability, and leakage of intracellular contents. The combined action of these mechanisms contributes to the potent antibacterial properties of ZIF-8 and its derivatives [38]. The enhanced antibacterial activity observed for CNS@ZIF-8 in the present study is likely associated with the synergistic contribution of these mechanisms together with the bioactive constituents present within the *R. cordatum*-derived carbon nanospheres.

#### 3.2.3. Time–Kill Curves Against MRSA

The bactericidal activities of CNS@ZIF-8 and ZIF-8 against MRSA were evaluated using a time–kill assay at 4, 8, and 24 h. Both formulations exhibited concentration- and time-dependent inhibitory effects on bacterial growth at 1 × MIC and 2 × MIC concentrations (Figure 8C).

At 4 h of incubation, treatment with CNS@ZIF-8 and ZIF-8 at 2 × MIC resulted in reductions of 2.21 and 1.95 log_10_ CFU/mL, respectively, compared with the untreated control group. As the incubation period progressed, antibacterial activity increased further. At 8 h, bacterial reductions of 2.80 and 2.22 log_10_ CFU/mL were observed for CNS@ZIF-8 and ZIF-8, respectively, at the same concentration. Following 24 h of incubation, CNS@ZIF-8 achieved a reduction of ≥3 log_10_ CFU/mL in viable bacterial counts, indicating bactericidal activity according to accepted microbiological criteria. In contrast, ZIF-8 treatment resulted in a 2.80 log_10_ CFU/mL reduction, suggesting substantial antibacterial activity but failing to reach the bactericidal threshold.

At the 1 × MIC concentration, both CNS@ZIF-8 and ZIF-8 demonstrated inhibitory effects against MRSA compared with the growth control. However, the reductions in bacterial counts were less pronounced than those observed at 2 × MIC, confirming a concentration-dependent antibacterial response. Representative colony images obtained after 24 h of incubation (Figure 8) further supported these findings, revealing a marked decrease in colony formation following CNS@ZIF-8 treatment compared with the untreated control.

Bioactive compound-loaded ZIF-8 systems have been reported to exhibit antibacterial activity. Meng et al. reported that ZIF-8 alone did not exhibit significant inhibitory activity against *S. aureus* after 24 h of incubation, whereas ZIF-8@CCM displayed pronounced and statistically significant antibacterial activity against the same bacterial strain [43]. Likewise, Soltani et al. investigated the time-dependent antibacterial efficacy of chloramphenicol-loaded ZIF-8 nanoparticles (CLN@ZIF-8). Their findings demonstrated that bare ZIF-8 nanoparticles achieved approximately 90% and 95% antibacterial activity after 6 and 24 h, respectively, whereas CLN@ZIF-8 exhibited markedly enhanced activity, resulting in more than 99.9% bacterial inhibition at both time points [44]. Consistent with these reports, the present study demonstrated that loading CNS into the ZIF-8 framework substantially improved antibacterial performance compared with ZIF-8 alone. Both 1 × MIC and 2 × MIC treatments produced progressive bacterial inhibition over time; however, the effect was more pronounced at 2 × MIC, where CNS@ZIF-8 achieved bactericidal activity after 24 h. The enhanced antibacterial efficacy observed for CNS@ZIF-8 may be attributed to the synergistic interaction between the intrinsic antimicrobial properties of ZIF-8 and the bioactive constituents present in the *Rheum cordatum*-derived carbon nanospheres. Furthermore, the gradual increase in bacterial inhibition over time suggests sustained antimicrobial activity, likely resulting from the controlled release characteristics and prolonged bacterial interaction provided by the ZIF-8 carrier system.

#### 3.2.4. Evaluation of Synergistic Effect of Zif-8 with CNS

The synergistic activity of ZIF-8 in combination with CNS against MRSA was assessed using the checkerboard assay (Table 3). The results revealed synergistic interactions between the two agents for both bacterial strains. Specifically, the ZIF-8–CNS combination exhibited fractional inhibitory concentration (FIC) index values of 0.50 for MRSA, confirming synergism. Previous studies support the synergistic antibacterial effects observed in the present study. Jamiri et al. reported that CS-PEG-G-10% DOX-4% ZIF-8 nanocomposite films exhibited pronounced synergistic antibacterial activity, particularly against *S. aureus* [45]. Similarly, the combination of copper oxide nanoparticles and anthraquinone-2-carboxylic acid has been shown to enhance antibacterial efficacy while potentially reducing the development of bacterial resistance [46]. Furthermore, studies on green-synthesized ZIF-8 nanoparticles have demonstrated that their combination with antimicrobial compounds improves antibacterial activity against a broad spectrum of pathogens, including multidrug-resistant (MDR) bacteria.

MIC and MBC values are expressed in μg/mL. MIC_A_ represents the MIC of ZIF-8 in combination with CNS, while MIC_B_ denotes the MIC of CNS in combination with ZIF-8. FIC_A_ and FIC_B_ correspond to the fractional inhibitory concentrations of ZIF-8 and CNS, respectively. FIC_i_ indicates the fractional inhibitory concentration index, which was used to determine the type of interaction between the ZIF-8 and CNS.

#### 3.2.5. Antibiofilm Activity Against MRSA

**Figure 9:**
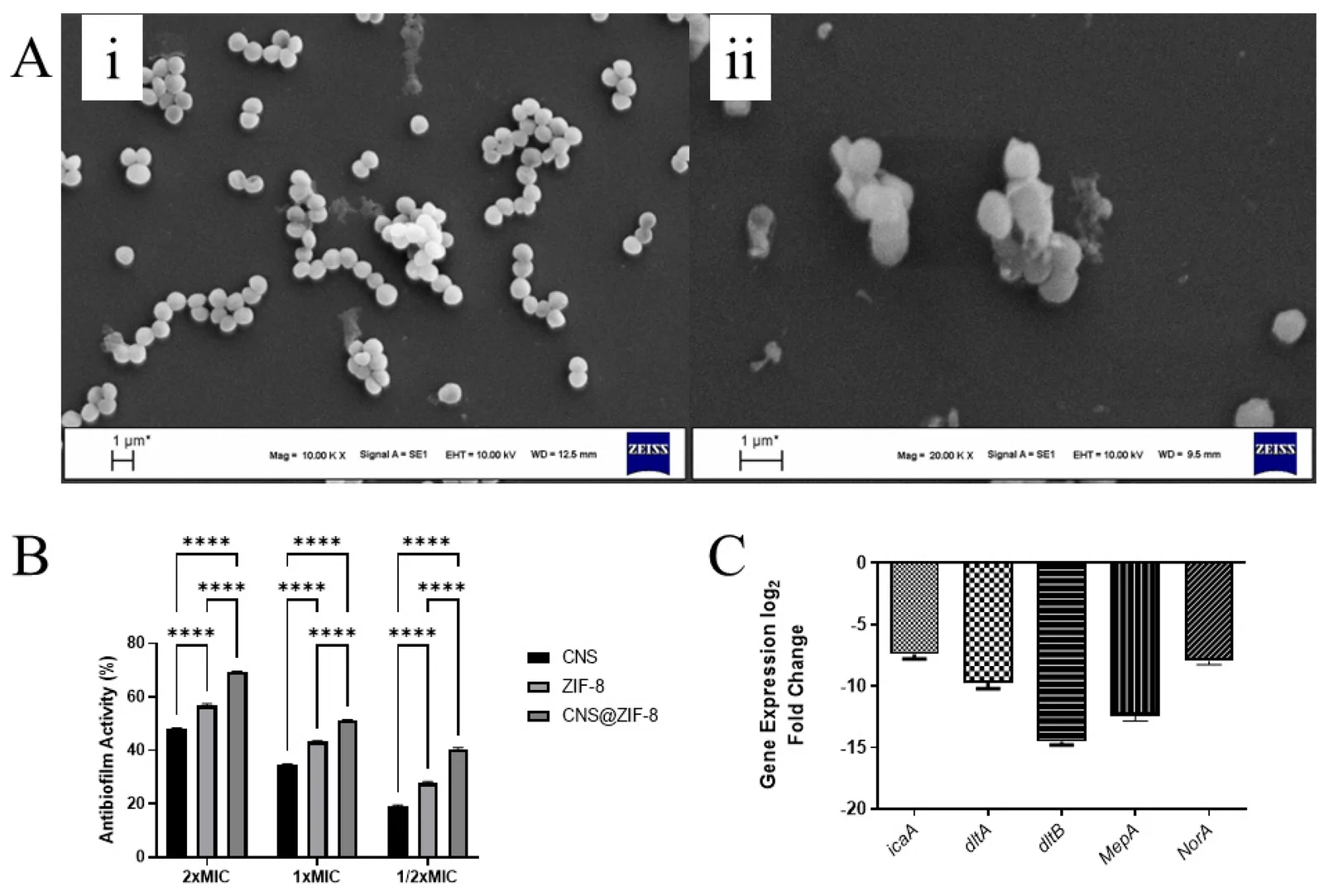
Antibiofilm activity, ultrastructural alterations, and modulation of virulence- and resistance-associated gene expression in MRSA following treatment with the CNS@ZIF-8 nanocomposite. Data are presented as mean ± SD from three independent experiments (n = 3). Statistical analysis was performed using one-way analysis of variance (ANOVA) followed by Tukey’s multiple comparisons test. **** *p* < 0.0001 was considered statistically significant. (**A**) SEM images of MRSA cells. (**i**), untreated control cells exhibiting intact spherical morphology, smooth cell surfaces, and normal cell clustering; (**ii**), MRSA cells treated with the CNS@ZIF-8 nanocomposite, showing severe morphological deformation, irregular cell surfaces, membrane damage, and disruption of normal cellular architecture, indicating antibacterial activity. (**B**) Antibiofilm activity of CNS, ZIF-8, and the CNS@ZIF-8 nanocomposite against (MRSA at 2 × MIC, 1 × MIC, and ½ × MIC concentrations, determined using the crystal violet biofilm assay. (**C**) Relative expression of the biofilm formation-, cell wall modification-, and efflux pump-associated genes (*icaA*, *dltA*, *dltB*, *mepA*, and *norA*) in MRSA following treatment with the CNS@ZIF-8 nanocomposite, determined by RT-qPCR)and expressed as log_2_ fold change relative to the untreated control. Negative log_2_ fold change values indicate downregulation of gene expression compared with the control.

The antibiofilm activities of CNS, ZIF-8, and CNS@ZIF-8 against the MRSA strain were evaluated at three different concentrations corresponding to 2 × MIC, 1 × MIC, and ½ × MIC. The obtained results demonstrated that all formulations inhibited biofilm formation in a concentration-dependent manner (Figure 9A).

The highest antibiofilm activity was observed at the 2 × MIC concentration. At this concentration, biofilm inhibition rates of 48.1 ± 0.25%, 56.6 ± 0.60%, and 69.3 ± 0.31% were recorded for CNS, ZIF-8, and CNS@ZIF-8, respectively. Among the tested formulations, CNS@ZIF-8 exhibited the strongest antibiofilm activity, indicating that incorporation of CNS into the ZIF-8 framework substantially enhanced its ability to suppress MRSA biofilm formation. At the MIC concentration, the inhibitory effect decreased compared with that observed at 2 × MIC; however, biofilm formation remained significantly suppressed. The inhibition rates at this concentration were determined to be 34.3 ± 0.45%, 43.1 ± 0.29%, and 51.1 ± 0.27% for CNS, ZIF-8, and CNS@ZIF-8, respectively. In contrast, all formulations exhibited lower antibiofilm activity at the ½ × MIC concentration, confirming the concentration-dependent nature of biofilm inhibition. Nevertheless, measurable inhibitory effects were still observed, suggesting that the tested formulations retained the ability to interfere with biofilm development even at sub-inhibitory concentrations.

The development of novel antimicrobial agents capable of preventing or disrupting MRSA biofilms has become an important focus of current research [47]. Previous studies have similarly highlighted the potent antibiofilm properties of ZIF-8-based nanomaterials. Tian et al. reported that ZIF-8 nanoparticles at a concentration of 200 µg/mL inhibited MRSA biofilm formation by more than 80% [48]. Likewise, Ehmud et al. demonstrated that ZIF-8 nanoparticles suppressed biofilm development by 99.3% at a concentration of 2.5 mg/mL [49]. In another study, a fucoidan-loaded FU@ZIF-L nanocomposite exhibited concentration-dependent antibiofilm activity, achieving 70.82 ± 0.58% inhibition at 100 µg/mL, with an IC_50_ value of 52.5 ± 1.05 µg/mL against MRSA [50]. Although direct comparisons between studies should be interpreted cautiously due to differences in bacterial strains, experimental conditions, and nanomaterial compositions, the findings consistently demonstrate the effectiveness of ZIF-based systems in controlling MRSA biofilms.

The enhanced antibiofilm activity observed for CNS@ZIF-8 may be attributed to the synergistic interaction between the bioactive constituents of *Rheum cordatum*-derived carbon nanospheres and the intrinsic antimicrobial properties of ZIF-8. In addition to its antibacterial activity, ZIF-8 has been reported to disrupt biofilm architecture through Zn^2+^ ion release, ROS generation, and direct interactions with the extracellular polymeric matrix, thereby reducing bacterial adhesion and biofilm stability.

The incorporation of CNS into the ZIF-8 framework may further enhance these effects by increasing the local concentration and availability of bioactive compounds at the biofilm interface.

Overall, CNS@ZIF-8 consistently demonstrated superior antibiofilm activity compared with free CNS and ZIF-8 alone at all tested concentrations. These findings suggest that the integration of CNS within the ZIF-8 nanocarrier system enhances its interaction with bacterial biofilms and improves its antibiofilm efficacy against MRSA. The pronounced inhibition observed at higher concentrations further indicates that CNS@ZIF-8 may be a promising strategy for controlling MRSA biofilm formation in vitro.

### 3.3. Evaluation of Bacterial Morphology

SEM was employed to examine the antibacterial effects of the CNS@ZIF-8 against MRSA. The SEM images demonstrated clear structural differences between treated and untreated control groups. After exposure to the compound at 2 × MIC concentration, a significant decrease in bacterial adhesion to the surface was observed. Moreover, pronounced morphological disruptions were evident in the treated bacterial cells. These structural alterations suggest that the antibacterial activity of the compound may be associated with damage to the bacterial cell envelope, indicating a potential loss of membrane integrity in MRSA following treatment (Figure 9B).

### 3.4. Gene Expression Analysis

Gene-expression analysis revealed that, following 24 h exposure to CNS@ZIF-8 at MIC concentration (128 µg/mL), all investigated target genes showed reduced expression relative to the untreated control. Analysis of normalized log_2_ fold-change values demonstrated that the most pronounced reduction was observed for *mepA* (log_2_FC = −1.026) followed by *dltA* (log_2_FC = −0.950), *dltB* (log_2_FC = −0.670), *icaA* (log_2_FC = −0.519), and *norA* (log_2_FC = −0.035) (Figure 9C).

Treatment with CNS@ZIF-8 resulted in variable downregulation of the investigated target genes in MRSA. The greatest reductions were observed for *mepA* and *dltA*, whereas *dltB* and *icaA* showed more moderate decreases in expression. In contrast, *norA* expression remained close to the control level, indicating that the transcriptional response to CNS@ZIF-8 differed among the investigated resistance- and biofilm-associated genes.

In the present study, treatment with the CNS@ZIF-8 nanocomposite resulted in pronounced downregulation of all investigated target genes in MRSA. The substantial suppression of *dltB*, *mepA*, and *dltA* expression suggests that the nanocomposite exerts effects beyond simple growth inhibition and may interfere with bacterial cell envelope integrity and resistance-associated pathways at the molecular level.

Previous studies have reported that ZIF-based nanocomposites exhibit potent antibacterial activity through multiple mechanisms. Guo et al. demonstrated that ZIF-based nanocomposites exert synergistic antibacterial effects against *Escherichia coli* and *Bacillus subtilis* and may increase membrane permeability by disrupting bacterial cell envelope integrity [51]. Similarly, ZIF-8 nanocomposites have been shown to possess significant antibacterial activity against both *S. aureus* and *E. coli*, while also reducing bacterial burden and accelerating wound healing in infected wound models [52,53]. These findings are in agreement with the results obtained in the present study and support the hypothesis that ZIF-based nanomaterials can affect bacterial physiology through mechanisms extending beyond direct growth inhibition.

Particularly noteworthy was the marked downregulation of *dltA* and *dltB*, genes involved in the D-alanylation of teichoic acids. The *dltABCD* operon plays a crucial role in regulating the surface charge of Gram-positive bacteria through the incorporation of D-alanine residues into teichoic acids. Suppression of this system may increase the negative surface charge of bacterial cells, thereby enhancing their susceptibility to cationic antimicrobial compounds and host defense peptides. The strong reduction in *dltA* and *dltB* expression observed in this study therefore suggests that CNS@ZIF-8 may compromise cell wall-associated resistance mechanisms in MRSA.

In addition, significant downregulation of the efflux pump-associated genes *norA* and *mepA* was observed following nanocomposite treatment. Multidrug efflux systems such as *norA* and *mepA* are known to contribute to antimicrobial resistance and bacterial adaptation to environmental stress by actively exporting toxic compounds from the cell [54]. Therefore, suppression of these genes may reduce the ability of MRSA to expel antimicrobial agents, potentially increasing intracellular accumulation of active compounds and enhancing antibacterial efficacy.

The observed decrease in *icaA* expression is consistent with modulation of biofilm-associated pathways following CNS@ZIF-8 exposure. This transcriptional finding is also in agreement with the reduced biofilm formation observed in the crystal-violet assay. The ica locus is involved in the synthesis of polysaccharide intercellular adhesin (PIA), an important component of the extracellular biofilm matrix that contributes to biofilm maturation and stability in *S. aureus*. Reduced *icaA* expression has previously been associated with impaired biofilm formation and decreased biofilm biomass. Therefore, the downregulation of *icaA* observed in the present study provides molecular evidence consistent with the antibiofilm phenotype of CNS@ZIF-8 [55].

Taken together, these findings suggest that CNS@ZIF-8 exerts a multifaceted antibacterial mechanism against MRSA by simultaneously targeting cell wall-associated defense systems, *mepA*-mediated multidrug efflux, and biofilm-related pathways. The coordinated downregulation of these virulence- and resistance-associated genes may contribute to the enhanced antibacterial and antibiofilm activities observed for the nanocomposite, highlighting its potential as a promising therapeutic strategy against MRSA infections.

### 3.5. Antioxidant Activity

The antioxidant properties of CNS, ZIF-8, and CNS@ZIF-8 formulations were evaluated by determining their abilities to scavenge DPPH and ABTS free radicals at different concentrations. To quantitatively compare the radical scavenging capacities of the tested formulations, IC_50_ values were calculated for each sample. The results obtained from both DPPH and ABTS assays revealed marked differences in the antioxidant performances of the investigated formulations (Table 4).

**Figure 10:**
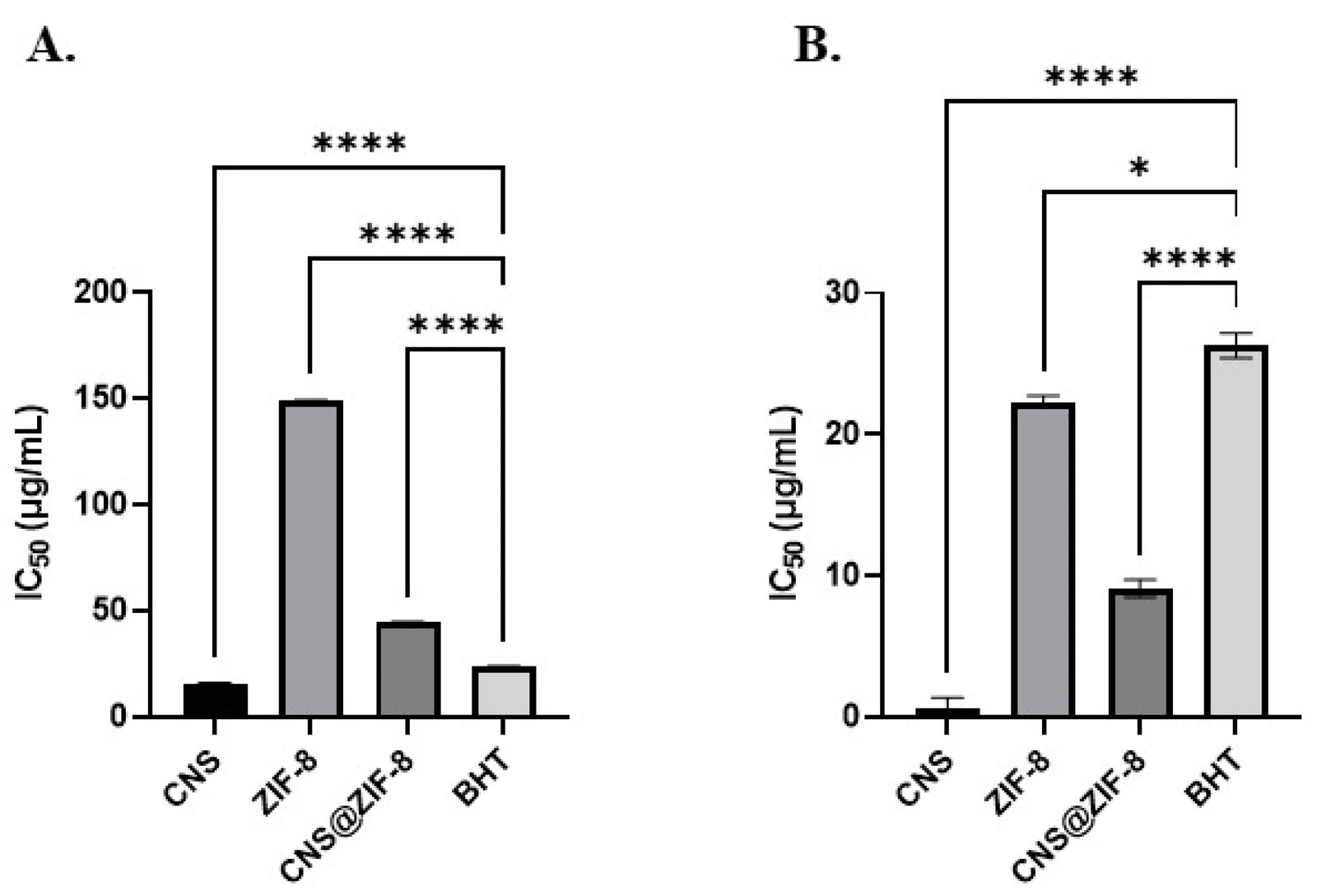
Antioxidant activity of CNS, ZIF-8, and the CNS@ZIF-8 nanocomposite evaluated by free radical scavenging assays. (**A**) DPPH radical scavenging activity expressed as IC_50_ (µg/mL) values. (**B**) ABTS radical scavenging activity expressed as IC_50_ (µg/mL) values. The antioxidant capacity of the synthesized materials was compared with the reference antioxidant BHT. Lower IC_50_ values indicate stronger free radical scavenging activity and higher antioxidant potential. Data are presented as the mean ± SD from three independent experiments (n = 3). Statistical analysis was performed using one-way analysis of variance (ANOVA) followed by Tukey’s multiple comparisons test. Differences were considered statistically significant at *p* < 0.05 (*) and *p* < 0.0001 (****).

In the DPPH radical scavenging assay, the IC_50_ values of CNS, ZIF-8, and CNS@ZIF-8 were determined to be 15.7 ± 0.23, 149.2 ± 0.32, and 45.1 ± 0.55 µg/mL, respectively. Among the tested formulations, CNS exhibited the strongest DPPH radical scavenging activity, as indicated by its lowest IC_50_ value, whereas ZIF-8 displayed the weakest antioxidant activity. The CNS@ZIF-8 nanocomposite demonstrated significantly improved antioxidant activity compared with ZIF-8 alone; however, its activity remained lower than that of free CNS. Similarly, in the ABTS radical scavenging assay, the IC_50_ values of CNS, ZIF-8, and CNS@ZIF-8 were found to be 0.62 ± 0.53, 22.3 ± 0.31, and 9.1 ± 0.44 µg/mL, respectively (Figure 10). Consistent with the DPPH results, CNS exhibited the highest ABTS radical scavenging capacity, followed by CNS@ZIF-8 and ZIF-8. The substantially lower IC_50_ values obtained in the ABTS assay compared with the DPPH assay indicate that the tested formulations were generally more effective in scavenging ABTS radicals.

Antioxidant compounds play a crucial role in protecting cells and tissues against oxidative damage by scavenging reactive oxygen species (ROS). Previous studies have demonstrated that the incorporation of bioactive phytochemicals into ZIF-8 structures can preserve or enhance their antioxidant properties. For instance, the CUR@ZIF-8 nanocomposite, containing curcumin encapsulated within the porous ZIF-8 framework, exhibited a DPPH radical scavenging IC_50_ value of 515.86 μg/mL, which was approximately 11-fold higher than that of free curcumin [56]. Similarly, Potnuru et al. developed a TIF@ZIF-8 nanocomposite by loading *Tamarindus indica* flower extract into ZIF-8 via a nanoprecipitation approach. The resulting nanocomposite displayed notable antioxidant activity, with IC_50_ values of 64.14 and 57.74 µg/mL in DPPH radical scavenging and lipid peroxidation inhibition assays, respectively [57]. The antioxidant potential of ZIF-8-based systems has been attributed to several factors. The high surface area and porous architecture of ZIF-8 facilitate the loading and stabilization of phytochemical compounds, thereby promoting interactions with free radicals [58]. Furthermore, zinc ions present within the framework have been associated with the regulation of oxidative stress and the activity of antioxidant defense systems. In addition, ZIF-8 can serve as a protective carrier that improves the stability and bioavailability of incorporated natural compounds while preserving their synergistic interactions [56,59].

In the present study, free CNS exhibited the strongest antioxidant activity in both the DPPH and ABTS assays. This finding suggests that the antioxidant capacity of CNS is largely attributable to the bioactive constituents present in the *Rheum cordatum*-derived carbon nanospheres. Phenolic and other antioxidant compounds associated with the carbon nanospheres may effectively neutralize DPPH and ABTS radicals through electron and/or hydrogen atom transfer. In contrast, incorporation of CNS into the ZIF-8 framework may partially restrict the accessibility of these active constituents and their diffusion into the reaction medium. Furthermore, the porous structure of ZIF-8 may influence the release of antioxidant compounds and their interaction with free radicals. These factors may account for the lower antioxidant activity observed for CNS@ZIF-8 compared with free CNS.

Phenolic constituents with antioxidant properties may indirectly enhance antibacterial performance through multiple mechanisms, including bacterial membrane disruption, interference with metabolic pathways, and inhibition of biofilm-associated processes. Therefore, the antioxidant capacity of the CNS@ZIF-8 nanocomposite can be considered an additional beneficial characteristic that may contribute to its overall biological functionality.

Overall, the results demonstrate that CNS possesses strong intrinsic antioxidant activity, likely due to the presence of bioactive phytochemical constituents derived from *Rheum cordatum*. Although incorporation into the ZIF-8 framework resulted in a partial reduction in antioxidant activity compared with free CNS, the CNS@ZIF-8 nanocomposite retained considerable radical scavenging capacity and exhibited markedly enhanced antioxidant performance relative to ZIF-8 alone. These findings suggest that the bioactive antioxidant components of CNS remain functionally active following incorporation into the ZIF-8 nanostructure.

### 3.6. Evaluation of Biocompatibility

**Figure 11:**
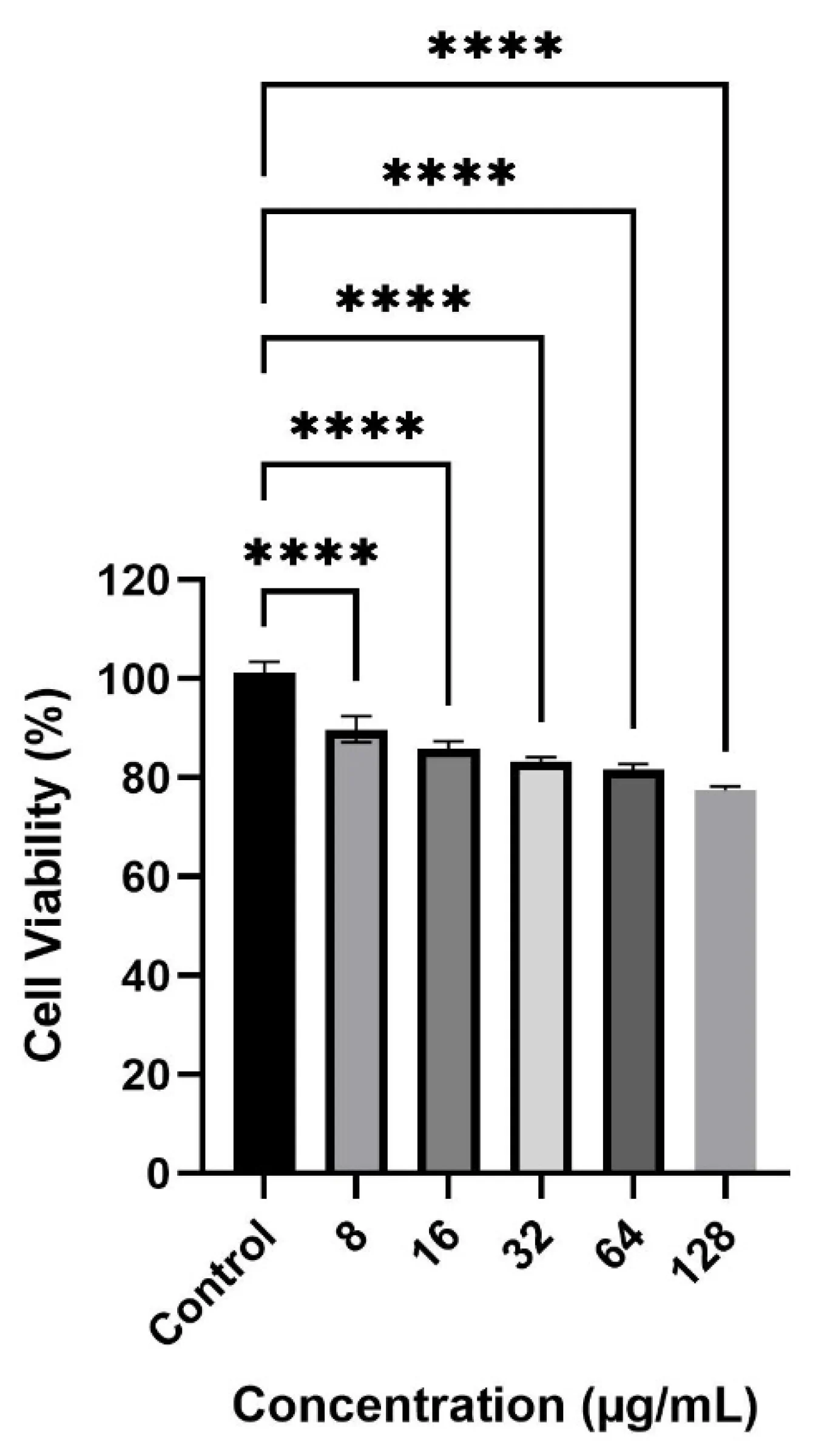
Cell viability results of NIH/3T3 cells exposed to (CNS@ZIF-8 at different concentrations (8–128 μg/mL). Cell viability was determined by MTT assay, and results are expressed as percentage of viable cells. Data are shown as mean ± SD (n = 3), **** *p* < 0.0001 is considered statistically significant.

The in vitro biocompatibility of the CNS@ZIF-8 nanocomposite was evaluated using the NIH/3T3 fibroblast cell line (Figure 11). In our study, high cell viability was observed across the entire tested concentration range of the CNS@ZIF-8 nanocomposite (8–128 μg/mL). At the lowest concentration tested (8 μg/mL), the cell viability was 89.7%, whereas a cell viability of 77.4% was observed at the highest concentration (128 μg/mL).

ZIF-8 may exhibit a low level of cytotoxicity, which could be associated with the intracellular concentrations of released Zn^2+^ ions and/or 2-MeIM ligands [60,61]. In contrast, the presence of CNSs may contribute to reducing the cytotoxic effects of the nanocomposite by modulating the interactions between ZIF-8 and cells. Furthermore, the ability of CNSs to limit the release of potentially toxic Zn^2+^ ions and/or 2-MeIM ligands may represent a possible mechanism underlying the enhanced biocompatibility observed for the CNS@ZIF-8 nanocomposite.

Overall, these findings indicate that the CNS@ZIF-8 nanocomposite does not exert a pronounced cytotoxic effect on NIH/3T3 fibroblast cells within the tested concentration range and exhibits good in vitro biocompatibility.

## 4. Conclusions

This study demonstrates the successful green synthesis of a CNS@ZIF-8 nanocomposite via the incorporation of *Rheum cordatum*-derived carbon nanospheres within a ZIF-8 framework. The nanocomposite showed the potent antibacterial and antibiofilm activities against MRSA and MSSA, along with notable antioxidant properties. Furthermore, mechanistic evaluation revealed that the antimicrobial activity of the nanocomposite is mediated through bacterial membrane damage, inhibition of biofilm formation, and downregulation of important virulence and antimicrobial resistance genes, including *dltA*, *dltB*, *norA*, *mepA* and *icaA*. Collectively, these findings demonstrate the potential of CNS@ZIF-8 as a multifunctional nanoplatform for the treatment of multidrug-resistant bacterial infections.

## Figures and Tables

**Table 1:** Primer sequences used for qRT-PCR analysis of virulence- and resistance-associated genes in MRSA. The genes *icaA*, *dltA*, *dltB*, *mepA*, and *norA* were selected to evaluate biofilm formation, cell wall modification, and efflux pump-mediated resistance, while *16S rRNA* was used as the internal reference gene for normalization of gene expression.

Genes	Primers
*icaA*	F: GGCTGCGGTAACTGGCAATCC R: CTTGCCAGTTAAAGATTGGGC
*dltB*	F: TGGTGGTTATTATTAGCGTGTTCG R: TGAATAGTCATGCCATCGTTATCG
*dltA*	F: AGTTATTGCTGTTGATGA R: CCCTGAAGTAAAGATGAT
*mepA*	F: GGCAAATAAAGGCCGTATGA R: CAGTCGCTTGAAGCATACCA
*norA*	F: ATCGGTTTAGTAATACCAGTCTTGC R: GCGATATAATCATTTGAGATAACGC
*16S rRNA*	F: GGACGGGTGAGTAATGTC R: TCTCAGACCAGCTAGGGATCG

**Table 2:** Comparison of the antibacterial activities of CNS, ZIF-8, the CNS@ZIF-8 nanocomposite, and the reference antibiotic vancomycin against MRSA and MSSA, expressed as MIC and MBC values (µg/mL).

Bacterial Strain	CNS		ZIF-8		CNS @ZIF-8		Vancomycin	
	MIC	MBC	MIC	MBC	MIC	MBC	MIC	MBC
MRSA	512	1024	256	512	128	256	2	4
MSSA	512	1024	512	1024	128	256	1	2

**Table 3:** Evaluation of the synergistic interaction between CNS and ZIF-8 against MRSA using the checkerboard microdilution assay.

Bacteria			MIC in Combination					
		MIC	MIC_A_	MIC_B_	FIC_A_	FIC_B_	FIC_i_	Interaction
MRSA	CNS	512	-	-	-	-	-	-
	ZIF-8	256	64	128	0.25	0.25	0.50	Synergistic

**Table 4:** IC_50_ (µg/mL) values of CNS, ZIF-8, and the CNS@ZIF-8 nanocomposite determined by DPPH and ABTS free radical scavenging assays. Lower IC_50_ values indicate higher antioxidant activity.

Compounds	DPPH	ABTS
CNS	15.7 ± 0.23	0.62 ± 0.53
ZIF-8	149.2± 0.32	22.3 ± 0.31
CNS@ZIF-8	45.1 ± 0.55	9.1 ± 0.44
BHT	23.4 ± 0.42	26.3 ± 0.63

## Data Availability

The datasets used and/or analyzed during the current study are available from the corresponding authors upon reasonable request.
